# Efficient synthetic protocols for the preparation of common N-heterocyclic carbene precursors

**DOI:** 10.3762/bjoc.11.252

**Published:** 2015-11-25

**Authors:** Morgan Hans, Jan Lorkowski, Albert Demonceau, Lionel Delaude

**Affiliations:** 1 Laboratory of Catalysis, Institut de Chimie (B6a), Allée du six Août 13, Quartier Agora, Université de Liège, 4000 Liège, Belgium; 2 Faculty of Chemistry, Adam Mickiewicz University in Poznań, Umultowska 89b, 61-614 Poznań, Poland

**Keywords:** cyclization, experimental procedure, imidazolinium salt, imidazolium salt, microwave heating

## Abstract

The one-pot condensation of glyoxal, two equivalents of cyclohexylamine, and paraformaldehyde in the presence of aqueous HBF_4_ provided a straightforward access to 1,3-dicyclohexylimidazolium tetrafluoroborate (ICy·HBF_4_). 1,3-Dibenzylimidazolium tetrafluoroborate (IBn·HBF_4_) was obtained along the same lines. To synthesize 1,3-diarylmidazolium salts, it was necessary to isolate the intermediate *N*,*N'*-diarylethylenediimines prior to their cyclization. Although this additional step required more time and reagents, it led to a much more efficient overall process. It also proved very convenient to carry out the synthesis of imidazolinium salts in parallel to their imidazolium counterparts via the reduction of the diimines into diammonium salts. The critical assembly of the C^2^ precarbenic unit was best achieved with paraformaldehyde and chlorotrimethylsilane in the case of imidazolium derivatives, whereas the use of triethyl orthoformate under microwave irradiation was most appropriate for the fast and efficient synthesis of imidazolinium salts. This strategy was applied to the synthesis of six common N-heterocyclic carbene precursors, namely, 1,3-dimesitylimidazolium chloride (IMes·HCl), 1,3-dimesitylimidazolium tetrafluoroborate (IMes·HBF_4_), 1,3-dimesitylimidazolinium chloride (SIMes·HCl), 1,3-bis(2,6-diisopropylphenyl)imidazolium chloride (IDip·HCl or IPr·HCl), 1,3-bis(2,6-diisopropylphenyl)imidazolinium chloride (SIDip·HCl or SIPr·HCl), and 1,3-bis(2,6-bis(diphenylmethyl)-4-methylphenyl)imidazolium chloride (IDip*·HCl or IPr*·HCl).

## Introduction

Since Arduengo and co-workers successfully isolated and characterized the first imidazol-2-ylidene derivative in 1991 [1–2], stable N*-*heterocyclic carbenes (NHCs) have become a staple of modern synthetic chemistry [3–7]. Over the past twenty five years, they have evolved from laboratory curiosities to ubiquitous ancillary ligands, not only for all the transition metals whether in high or low oxidation state [8–10], but also for lanthanides and actinides [11–12], as well as for main group elements [12–13]. Countless applications in homogeneous catalysis have already taken advantage of the remarkable stereoelectronic properties and structural diversity of these organometallic species [14–17]. To give just a single example, NHC ligands played a crucial role in the development of highly efficient ruthenium initiators for olefin metathesis and related reactions [18–21]. Lately, these divalent carbon species have also emerged as powerful nucleophilic organocatalysts for polymer chemistry [22–23] and organic synthesis [24–26]. In particular, they were successfully employed for the umpolung of carbonyl compounds, sometimes in an asymmetric fashion [27–29].

**Scheme 1:**
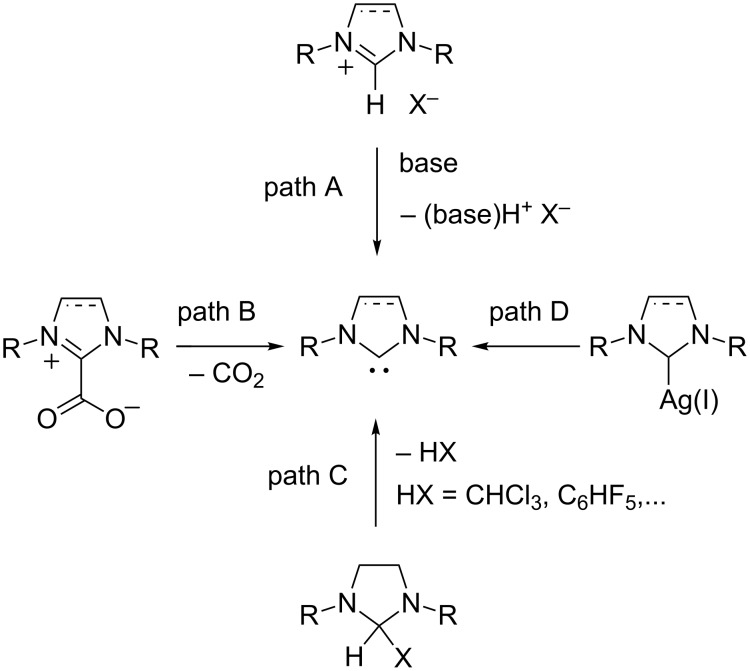
Various synthetic paths leading to the formation of NHCs.

Currently, the NHCs most frequently encountered are based on the imidazol-2-ylidene and imidazolin-2-ylidene scaffolds, which are easily accessible via the deprotonation of imidazolium or imidazolinium salts with a strong base (Scheme 1, path A) [26,30]. The reaction is often carried out in situ to avoid the isolation of air- and moisture-sensitive free carbenes. Thus, the mixture of an imidazol(in)ium salt and a base serves de facto as a carbene source for most catalytic and synthetic purposes. Alternative methods to generate NHCs without the intervention of a base, which might lead to unwanted side-reactions, include the facile cleavage of NHC·CO_2_ zwitterions (Scheme 1, path B) [31–35], the thermolysis of labile imidazolidine adducts (Scheme 1, path C) [36–38], or the recourse to Ag(I)–NHC complexes as NHC delivery agents (Scheme 1, path D) [39–40]. In many cases, however, these NHC surrogates are obtained from azolium intermediates. Hence, imidazolium and imidazolinium salts are the most common NHC precursors and their synthesis from acyclic starting materials is of utmost practical importance [41].

**Scheme 2:**
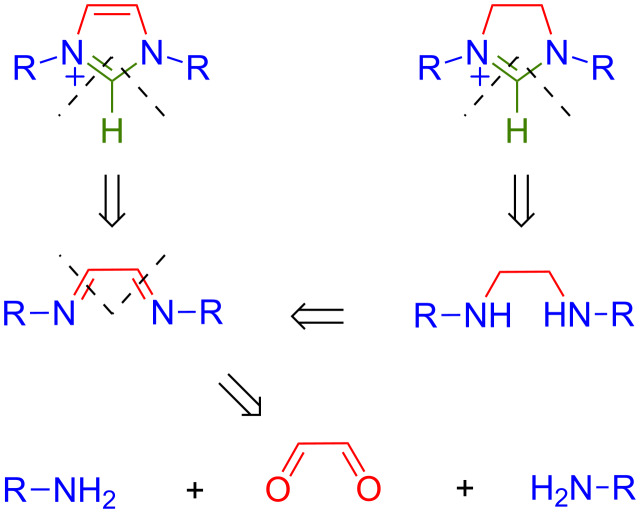
Retrosynthetic path for the preparation of symmetrical imidazolium and imidazolinium salts from simple acyclic precursors.

One of the most atom-economical and straightforward path to elaborate symmetrical imidazolium salts involves the combination of glyoxal, which provides the C^4^–C^5^ heterocyclic backbone of the NHC, two equivalents of a primary alkylamine or aniline, to introduce the N^1^ and N^3^ modular units, and a suitable C_1_ building block for joining the precarbenic C^2^ center (Scheme 2). An additional reduction of the intermediate diimines into diamines is required prior to the assembly of the corresponding imidazolinium derivatives. The first embodiment of this general strategy dates back to 1991 when Arduengo patented the one-pot condensation of glyoxal, two equivalents of an amine, and paraformaldehyde in the presence of hydrochloric acid to afford 1,3-disubstituted imidazolium chlorides [42]. Although this procedure was rather efficient when applied to simple alkylamines, its extension to the synthesis of 1,3-diarylimidazolium salts usually failed due to the formation of dark, tarry ionomer byproducts that could only be painstakingly separated from the desired compounds, thereby leading to low yields of tainted products [43]. This practical complication was very unfortunate because bulky aromatic substituents, such as mesityl (2,4,6-trimethylphenyl) or 2,6-diisopropylphenyl groups, often provide the right balance of electronic donation and steric protection to many NHC-based catalytic systems. Accordingly, it spurred sustained research efforts to improve and optimize experimental conditions leading to imidazol(in)ium salts in both academic [43–45] and industrial laboratories [46]. Because of the incremental nature of these endeavors, a large number of valuable synthetic procedures have been scattered in the literature, often relegated to supporting information, and comparison of their respective merits has become more and more challenging.

**Figure 1:**
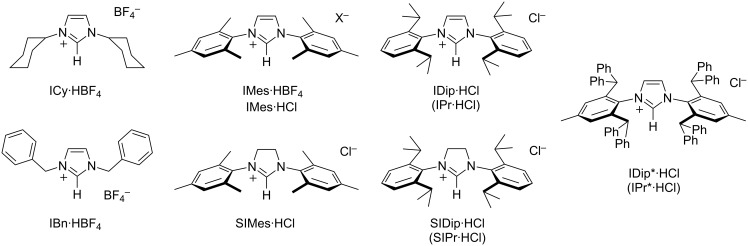
Structures of the imidazolium and imidazolinium salts discussed in this study and their acronyms.

In this report, we aimed at collecting a series of efficient synthetic protocols for the preparation of eight common N-heterocyclic carbene precursors differing by the nature of their central core (imidazolium or imidazolinium), the choice of the associated counterion (chloride or tetrafluoroborate), or the steric bulk of their nitrogen substituents (ranging from small, flexible cyclohexyl rings to hefty 2,6-bis(diphenylmethyl)-4-methylphenyl groups, Figure 1). For each target compound, we strove to put together the most straightforward, detailed experimental procedure that was checked to afford high yield and purity, and a full characterization by ^1^H and ^13^C NMR spectroscopies.

## Results and Discussion

### Synthesis of 1,3-dicylohexylimidazolium tetrafluoroborate

**Scheme 3:**
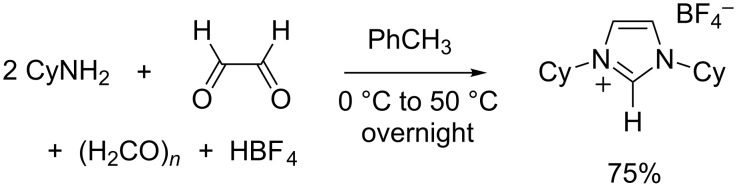
Synthesis of 1,3-dicyclohexylimidazolium tetrafluoroborate (ICy·HBF_4_).

The one-pot synthesis of 1,3-dicyclohexylimidazolium chloride (ICy·HCl) was first disclosed in the open literature by Herrmann and co-workers in 1996 [47]. The reaction proceeded smoothly and the product did not show any tendency to form an ionic liquid, unlike its lower weight unsymmetrical analogues. It turned out, however, to be highly hygroscopic, which hindered its purification and subsequent reactions with moisture-sensitive bases or organometallic compounds. Other counterions were found to alleviate this tendency. In particular, the replacement of HCl with aqueous HBF_4_ in the original procedure allowed us [48] and others [49] to isolate ICy·HBF_4_ as a well-behaved, non-hygroscopic solid that could be easily purified by recrystallization from isopropanol. Typical yields were in the 70–80% range (Scheme 3).

### Synthesis of 1,3-dibenzylimidazolium tetrafluoroborate

**Scheme 4:**
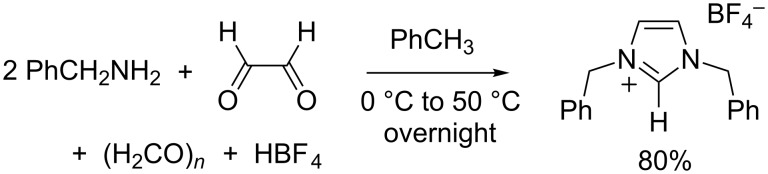
Synthesis of 1,3-dibenzylimidazolium tetrafluoroborate (IBn·HBF_4_).

At first sight, benzyl chloride or benzyl bromide seem to be ideal candidates to prepare 1,3-dibenzylimidazolium salts via a double alkylation of imidazole. Indeed, these primary alkyl halides are highly reactive toward nucleophiles and do not undergo elimination reactions. Accordingly, numerous procedures were reported for the two-step synthesis of 1,3-dibenzylimidazolium halides via the formation of 1-benzylimidazole [50–53]. In our hands, however, the quaternization of this intermediate with benzyl chloride or bromide was often sluggish, which led to incomplete conversions and residues of unpleasant, lachrymatory reagents. We were very pleased to find out that the one-pot procedure described above for 1,3-dicyclohexylimidazolium tetrafluoroborate could be seamlessly translated to the preparation of 1,3-dibenzylimidazolium tetrafluoroborate (IBn·HBF_4_) from glyoxal, benzylamine, paraformaldehyde, and tetrafluoroboric acid (Scheme 4). To the best of our knowledge, this superior route had not been explored so far.

### Synthesis of 1,3-dimesitylimidazolium salts

Although little experimental details were supplied, the preparation of 1,3-dimesitylimidazolium chloride (IMes·HCl) was first disclosed by Arduengo et al. in 1992 using a one-pot procedure [54]. Several research groups noticed that this strategy often led to dark brown molasses out of which a solid product could only be painstakingly extracted after extensive washing, resulting in low yields of rather impure materials [43,55–56]. To circumvent this problem, Arduengo and co-workers devised a two-step protocol involving the isolation of *N,N'*-dimesitylethylenediimine followed by cyclization with chloromethyl ethyl ether, which slowly reacted to afford both the C^2^ imidazolium center and the chloride counterion [43]. While this procedure remained low-yielding and time-consuming, it significantly eased the isolation of the final product, which cleanly precipitated from the reaction mixture. Most importantly, this work demonstrated the importance of isolating the intermediate Schiff base prior to its cyclization, a feature that proved crucial to successfully achieve the synthesis of 1,3-diarylimidazolium salts by late introduction of the precarbenic atom moiety. It should be pointed out that Nolan et al. also reached this conclusion when they first optimized the synthesis of 1,3-bis(2,6-diisopropylphenyl)imidazolium chloride in 1999 (vide infra) [57].

**Scheme 5:**
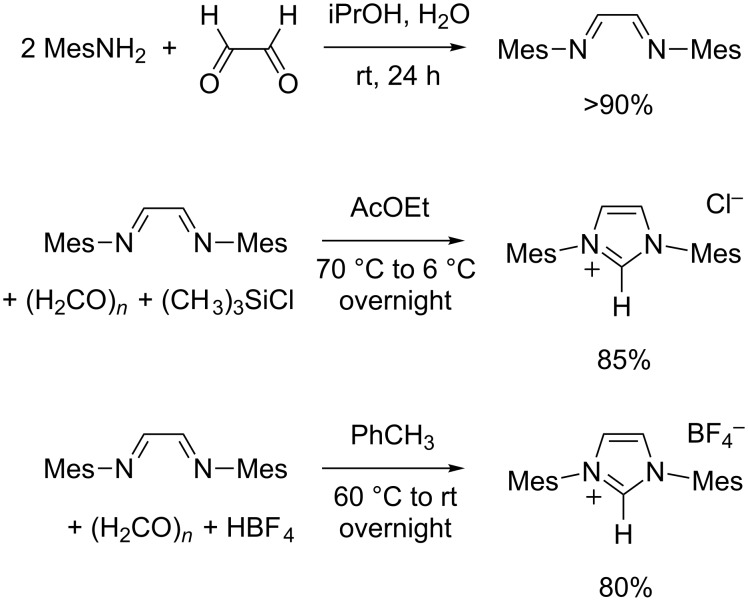
Synthesis of 1,3-dimesitylimidazolium salts (IMes·HCl and IMes·HBF_4_).

Over the years, several variations were reported on the two-step route leading to 1,3-dimesitylimidazolium chloride [41]. Only minor changes concerned the initial condensation between glyoxal and two equivalents of mesitylamine (Scheme 5). The reaction proceeds readily in aqueous/alcoholic mixtures at room temperature and the product begins to separate as a bright yellow solid after a few minutes. Of note, second and even third crops of precipitate are usually obtained upon work-up during the synthesis of diimines. They may be added to the first crop in order to further increase the yield, but their purity needs to be checked beforehand. Formic acid is sometimes added as a catalyst but does not seem to be mandatory, maybe because glyoxal is often contaminated with glyoxylic and oxalic acids, especially upon prolonged storage under aerobic conditions. More significant alterations were brought to the cyclization step. In addition to the use of chloromethyl ethyl ether pioneered by Arduengo et al. [43], mixtures of paraformaldehyde and HCl in anhydrous solvents were investigated by Bantreil and Nolan [45], while Hintermann identified chlorotrimethylsilane as a convenient source of chloride counterions [44]. Furthermore, this last reagent does not lead to the formation of water, which can hydrolyze the starting diimine and has a deleterious influence on the reaction course. Thus, we adopted the experimental procedure carefully optimized by Hintermann to obtain IMes·HCl in ca. 85% yield (Scheme 5).

We were also interested in the preparation of IMes·HBF_4_ because imidazolium tetrafluoroborates are usually less hygroscopic and easier to crystallize than chlorides [45]. Moreover, when imidazolium salts are used to generate NHCs in situ, for instance to accomplish organocatalytic transformations, the exact nature of the counterion may influence the solubility and the deprotonation rate of the carbene precursor [58]. A report from 2010 had shown that treating *N,N'*-dimesitylethylenediimine with paraformaldehyde and a 48% aqueous solution of tetrafluoroboric acid in warm toluene afforded 1,3-dimesitylimidazolium tetrafluoroborate without any apparent complication [59]. We have further optimized this procedure on the occasion of a mechanistic study of the Staudinger reaction catalyzed by NHC·ketene zwitterions (Scheme 5) [60].

### Synthesis of 1,3-dimesitylimidazolinium chloride

**Scheme 6:**
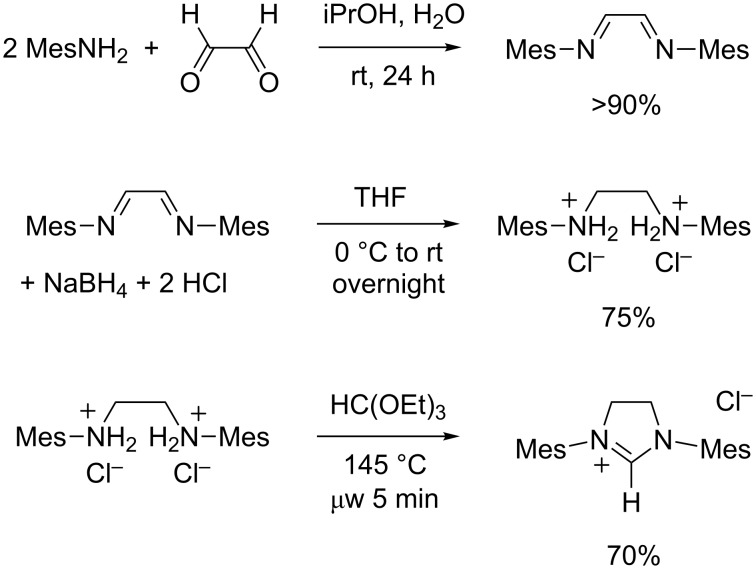
Synthesis of 1,3-dimesitylimidazolinium chloride (SIMes·HCl).

The “saturated” analogue of the IMes carbene, 1,3-dimesitylimidazolin-2-ylidene (SIMes), was first isolated in 1995 by Arduengo et al. [61] who later disclosed the experimental details of the synthetic path leading to this stable NHC and its immediate precursor, 1,3-dimesitylimidazolinium chloride (SIMes·HCl) [43]. The latter salt was obtained in three steps starting from widely available, acyclic reagents (Scheme 6). First, the condensation of glyoxal with two equivalents of mesitylamine afforded the corresponding diimine, as described above for the synthesis of IMes·HCl and IMes·HBF_4_ (cf. Scheme 5). Next, the diimine was reduced into a diamine with sodium borohydride in THF, followed by an acidic work-up with aqueous hydrochloric acid to quench the excess of hydride and to precipitate *N*,*N'*-dimesitylethylenediammonium dichloride as a stable white solid. A third step afforded the final heterocyclic product upon ring-closure with triethyl orthoformate in the presence of a catalytic amount of formic acid. In this reaction, the orthoester served both as a solvent and a precarbenic C^2^ provider.

Following the seminal contribution of Arduengo and co-workers, several other research groups proposed experimental procedures for the preparation of imidazolinium salts from *N*,*N'*-disubstituted 1,2-ethanediamines or their ammonium salts and triethyl orthoformate [62–65]. In most cases, prolonged heating under reflux conditions was necessary to reach satisfactory conversions, even when ethanol was distilled off the reaction mixture to drive the equilibrium toward completion. In 2006, we found that microwave irradiation allowed to dramatically reduce the reaction time from hours to minutes, while affording very high yields of pure products [66]. We have applied this procedure to the synthesis of a wide range of cyclic amidinium salts differing by their ring size, N-substituents, and counterions [67]. In 2010, we have further optimized the microwave-assisted synthesis of SIMes·HCl to turn it into a convenient, laboratory-scale preparation [68]. With the latest implementation of our protocol, which uses an 80 mL glass vessel in a monomodal microwave reactor, it took 5 minutes to perform the cyclization on a 50 mmol batch and the product was isolated in 70% yield after purification (Scheme 6).

### Synthesis of 1,3-bis(2,6-diisopropylphenyl)imidazolium chloride

Huang and Nolan first reported the introduction of 2,6-diisopropylphenyl groups on an imidazolylidene backbone in 1999 while searching for bulky NHC ligands to coordinate onto palladium catalysts for Kumada cross-coupling reactions [57]. The resulting carbene was nicknamed IPr and this designation still persists in the literature, although IDip is a more fitting acronym to avoid any confusion with 1,3-di(isopropyl)imidazol-2-ylidene. Thus, 1,3-bis(2,6-diisopropylphenyl)imidazolium chloride (IDip·HCl) was obtained following a two-step procedure that first involved the condensation of glyoxal and two equivalents of 2,6-diisopropylaniline into the corresponding diazabutadiene. This intermediate was then cyclized into the final product using paraformaldehyde in toluene as the precarbenic C^2^ donor reagent and anhydrous HCl in dioxane as the source of the counterion. Soon thereafter, Arduengo and co-workers described the synthesis of IDip·HCl using chloromethyl ethyl ether as a single provider for the azolium chloride building block [43]. From a practical point of view, both procedures represented a significant breakthrough, because the one-pot strategy was largely inefficient for imidazolium salts bearing bulky aromatic substituents on their nitrogen atoms.

**Scheme 7:**
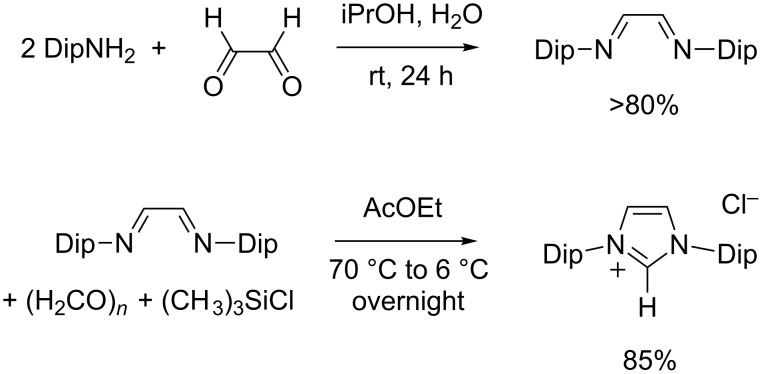
Synthesis of 1,3-bis(2,6-diisopropylphenyl)imidazolium chloride (IDip·HCl).

Over the years, minor changes were brought to the original protocols of Nolan and Arduengo in order to improve yields that did not initially pass the 50% threshold [45,69–70]. Yet, the most convenient preparation available to date for IDip·HCl was proposed in 2007 by Hintermann [44]. It involved the reaction of *N*,*N'*-bis(2,6-diisopropylphenyl)ethylenediimine with paraformaldehyde and chlorotrimethylsilane in ethyl acetate (Scheme 7). In our hands, this procedure proved reliable and afforded typical yields of 85%. Its major drawback is the necessity to work under high dilution conditions, which implies the use of large amounts of solvent and hinders scale up.

### Synthesis of 1,3-bis(2,6-diisopropylphenyl)imidazolinium chloride

**Scheme 8:**
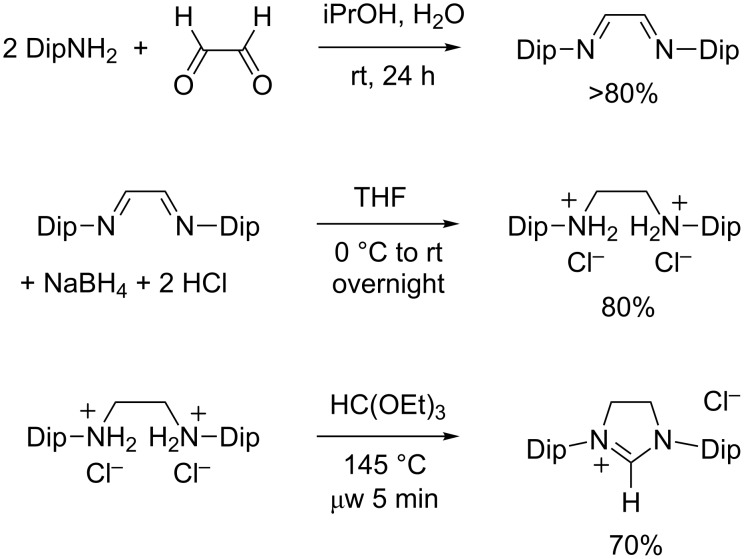
Synthesis of 1,3-bis(2,6-diisopropylphenyl)imidazolinium chloride (SIDip·HCl).

In most cases, experimental procedures leading to 1,3-dimesitylimidazolinium salts could be successfully extended to their 1,3-bis(2,6-diisopropylphenyl) counterparts without any adaptation. Thus, the three-step synthesis of SIDip·HCl (also known as SIPr·HCl) initially reported by Arduengo et al. closely matched the one defined for SIMes·HCl in terms of experimental conditions and yields [43]. Likewise, our microwave-assisted cyclization performed equally well when applied to *N*,*N'*-bis(2,6-diisopropylphenyl)ethylenediammonium chloride instead of the dimesityl intermediate (Scheme 8). In both cases, the desired product was isolated in (70 ± 5)% yield after a very simple work-up that involved filtration and washing.

### Synthesis of 1,3-bis(2,6-bis(diphenylmethyl)-4-methylphenyl)imidazolium chloride

**Scheme 9:**
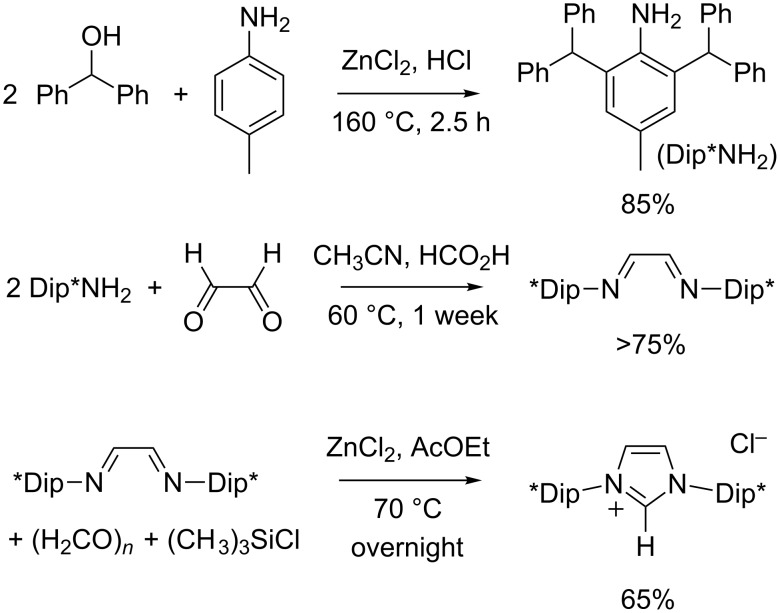
Synthesis of 1,3-bis(2,6-bis(diphenylmethyl)-4-methylphenyl)imidazolium chloride (IDip*·HCl).

In 2010, the group of Markó designed a very bulky, yet flexible NHC ligand by replacing the methyl groups of IDip with phenyl rings [71]. This new highly hindered carbene that we shall designate as IDip* (but it is also trivially named IPr*) was readily obtained by deprotonation of 1,3-bis(2,6-bis(diphenylmethyl)-4-methylphenyl)imidazolium chloride (IDip*·HCl). The synthesis of this stable precursor was accomplished in three steps starting from commercially available reagents (Scheme 9). First, *p*-toluidine was dialkylated with diphenylmethanol (benzhydrol) in the presence of stoichiometric amounts of HCl and ZnCl_2_. This Friedel–Crafts alkylation was carried out under solvent-free conditions and afforded high yields of the bulky aniline needed to follow the Arduengo formylative cyclization path. It was originally performed in a sealed tube under autogeneous pressure at 160 °C. We checked that the reaction could be carried out in an open vessel without any detrimental consequence, thereby leading to a safer experimental procedure. In the second step, 2,6-bis(diphenylmethyl)-4-methylaniline (Dip*NH_2_) was reacted with aqueous glyoxal to form the corresponding ethylenediimine. Markó et al. performed this condensation in dichloromethane containing formic acid as a catalyst and anhydrous magnesium sulfate as a dehydrating agent. We found this procedure difficult to reproduce. Moreover, a rather tedious work-up was required to separate and to purify the product. Inspired by a report from Cole and co-workers on the preparation of another bulky imidazolium salt [72], we found that acetonitrile was a much more convenient solvent than dichloromethane to achieve the condensation of Dip*NH_2_ and glyoxal. Although the reaction was slow and took about a week to reach completion at 60 °C, the desired diazabutadiene cleanly precipitated from the reaction mixture and could be isolated in high yield by simple filtration and washing.

For the critical cyclization step of IDip*·HCl, Markó et al. ingeniously took advantage of the Lewis acidity of zinc chloride to activate paraformaldehyde and of its coordinating ability to maintain the intermediate diimine in the required *s-cis* conformation [71]. Concentrated hydrochloric acid was added as the counterion source and the final imidazolium product was isolated in 50–60% yield. We further improved this procedure through the use of chlorotrimethylsilane as the chloride donor to minimize hydrolysis and other side-reactions of the diimine. The templating effect of ZnCl_2_ was also maximized by combining this stoichiometric additive with the diimine and paraformaldehyde prior to the addition of Me_3_SiCl. Under these revised conditions, IDip*·HCl was isolated in 65% yield after recrystallization.

## Conclusion

The one-pot condensation of glyoxal, two equivalents of a primary alkylamine, and paraformaldehyde in the presence of aqueous HBF_4_ provided a straightforward access to symmetrical 1,3-dialkylimidazolium tetrafluoroborates. To achieve the preparation of 1,3-diarylimidazolium salts, it was necessary to isolate the intermediate diimines prior to their cyclization. Although this additional step required more time and reagents, it led to a much more efficient overall process. It proved also very convenient to carry out the synthesis of imidazolinium salts in parallel to their imidazolium counterparts via the reduction of the diimines into diamines or diammonium salts. The critical assembly of the C^2^ precarbenic unit was best achieved with paraformaldehyde and chlorotrimethylsilane in the case of the imidazolium derivatives, whereas the use of triethyl orthoformate under microwave irradiation was most appropriate for the fast and efficient synthesis of imidazolinium salts.

With the possible exception of a monomodal microwave reactor, all the equipment and glassware needed to carry out the syntheses outlined in this report are widely available in chemical laboratories and do not require any particular skills from the experimenter. Furthermore, the detailed experimental procedures supplied in Supporting Information File 1 of this article are easy to scale up or down according to the particular needs for a given compound. Thus, we hope that they will be helpful to the large community of organic and organometallic chemists working with NHCs.

## Supporting Information

File 1 Full experimental section with detailed synthetic procedures and analytical data for all the compounds.
