# Supplementary material for: Efficient synthetic protocols for the preparation of common N-heterocyclic carbene precursors
Source: Beilstein J Org Chem. 2015 Nov 25;11:2318–25. doi: 10.3762/bjoc.11.252 (PMC4685873; doi:10.3762/bjoc.11.252)
Supplement: File 1 — Full experimental section with detailed synthetic procedures and analytical data for all the compounds. [file Beilstein_J_Org_Chem-11-2318-s001.pdf]

Supporting Information

for

Efficient synthetic protocols for the preparation of common N-heterocyclic carbene precursors

Morgan Hans[1], Jan Lorkowski[1,2], Albert Demonceau[1] and Lionel Delaude[*1]

Address: [1]Laboratory of Catalysis, Institut de Chimie (B6a), Allée du six Août 13, Quartier Agora, Université de Liège, 4000 Liège, Belgium and [2]Faculty of Chemistry, Adam Mickiewicz University in Poznań, Umultowska 89b, 61-614 Poznań, Poland

E-mail: Lionel Delaude - L.Delaude@ulg.ac.be

*Corresponding author

Full experimental section with detailed synthetic procedures and analytical data for all the compounds

Table of contents

1. General information ... s3
2. Synthesis of 1,3-dicyclohexylimidazolium tetrafluoroborate (ICy·HBF_4) ... s4
3. Synthesis of 1,3-dibenzylimidazolium tetrafluoroborate (IBn·HBF_4) ... s6
4. Synthesis of *N,N′*-dimesitylethylenediimine ... s8
5. Synthesis of 1,3-dimesitylimidazolium chloride (IMes·HCl) ... s10
6. Synthesis of 1,3-dimesitylimidazolium tetrafluoroborate (IMes·HBF_4) ... s12
7. Synthesis of *N,N′*-dimesitylethylenediamine dihydrochloride ... s14
8. Synthesis of 1,3-dimesitylimidazolinium chloride (SIMes·HCl) ... s16
9. Synthesis of *N,N′*-bis(2,6-diisopropylphenyl)ethylenediimine ... s18
10. Synthesis of 1,3-bis(2,6-diisopropylphenyl)imidazolium chloride (IDip·HCl) ... s20
11. Synthesis of *N,N′*-bis(2,6-diisopropylphenyl)ethylenediamine dihydrochloride ... s22
12. Synthesis of 1,3-bis(2,6-diisopropylphenyl)imidazolinium chloride (SIDip·HCl) ... s24
13. Synthesis of 2,6-bis(diphenylmethyl)-4-methylaniline ... s26
14. Synthesis of *N,N′*-bis(2,6-bis(diphenylmethyl)-4-methylphenyl)ethylenediimine ... s28
15. Synthesis of 1,3-bis(2,6-bis(diphenylmethyl)-4-methylphenyl)imidazolium chloride (IDip*·HCl) ... s30
16. References and notes ... s32

1. General information

All the syntheses were carried out under a normal atmosphere without any precaution to avoid oxygen and moisture. Solvents were purchased from Labotec and used as received. All the other chemicals were obtained from Aldrich and used as received. Glyoxal (40% aqueous solution) was stored in a cold room at +6 °C. Microwave-assisted syntheses were carried out using a CEM Discover instrument. Melting points were recorded with an Electrothermal apparatus and are not corrected. Unless otherwise specified, ^{1}H and ^{13}C NMR spectra were recorded at 298 K with a Bruker DRX 400 spectrometer operating at 400.13 and 100.62 MHz, respectively. Chemical shifts are listed in parts per million downfield from TMS and are referenced to solvent peaks or TMS. Assignments were established with the help of APT, COSY, HMBC, and HSQC sequences.

Abbreviations used:

i	*ipso*
o	*ortho*
m	*meta*
p	*para*
br	broad
s	singlet
d	doublet
t	triplet
m	multiplet
sept	septuplet
Bn	benzyl
Cy	cyclohexyl
Dip	2,6-diisopropylphenyl
Dip*	2,6-bis(diphenylmethyl)-4-methylphenyl
Im	imidazolium or imidazolinium
Mes	mesityl (2,4,6-trimethylphenyl)
Ph	phenyl

2. Synthesis of 1,3-dicyclohexylimidazolium tetrafluoroborate (ICy·HBF_4)

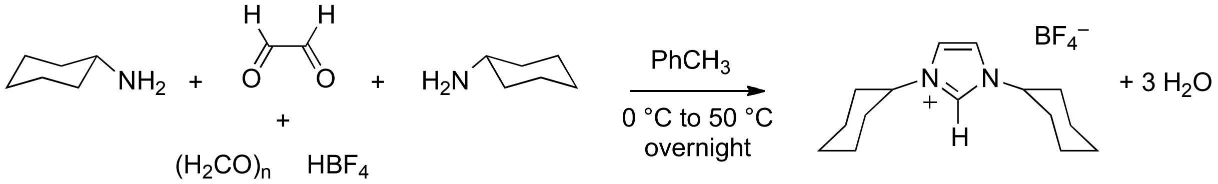

MF	$C_6H_{13}N$	$(CH_2O)_n$	$C_2H_2O_2$	HBF_4	$C_{15}H_{25}BF_4N_2$
MW	99.1754	30.0262	58.0366	87.8125	320.1805
amount (mmol)	2 × 100	100	100	125	100
mass (g)	2 × 9.9175	3.0026	14.51 g of 40% aq. sln	22.87 g of 48% aq. sln	32.0181 (100% yield)

Experimental procedure: A 500 mL round-bottom flask equipped with a magnetic stirring bar and a stopper was charged with cyclohexylamine (9.92 g, 100 mmol), toluene (100 mL), and paraformaldehyde (3.00 g, 100 mmol). The resulting milky suspension was stirred for 30 min at room temperature until the solid was almost completely dissolved. The mixture was cooled to 0 °C in an ice/water bath and a second equivalent of cyclohexylamine (9.92 g, 100 mmol) was added. The resulting yellow solution was stirred for 10 min at 0 °C before tetrafluoroboric acid (48% aqueous solution, 22.87 g, 16.3 mL, 125 mmol) was added portionwise in 15 min. The cooling bath was removed and glyoxal (40 % aqueous solution, 14.51 g, 11.5 mL, 100 mmol) was added. The resulting cloudy mixture was stirred overnight (ca. 12 h) in an oil bath at 50 °C. After cooling to room temperature, dichloromethane (100 mL) and water (50 mL) were added and the layers were separated. The aqueous phase was extracted with 3 portions of dichloromethane (100 mL each). The organic layers were combined and dried over $MgSO_4$. After evaporation of the solvent, the beige residue was recrystallized from isopropanol to afford pure 1,3-dicyclohexylimidazolium tetrafluoroborate as white needles (typical yield: 24 g, 75%).

Analytical data: Mp 171–172 °C. ^1H NMR (400 MHz, $CDCl_3$): δ = 8.92 (s, 1 H, CH^2 Im), 7.47 (s, 2 H, $CH^{4,5}$ Im), 4.35–4.29 (m, 2 H, NC*H*), 2.19–2.16 (m, 4 H, Cy), 1.92–1.89 (m, 4 H, Cy), 1.74–1.67 (m, 6 H, Cy), 1.52–1.42 (m, 4 H, Cy), 1.33–1.25 (m, 2 H, Cy) ppm. ^{13}C NMR (100 MHz, $CDCl_3$): δ = 133.0 (CH^2 Im), 120.5 ($CH^{4,5}$ Im), 60.1 (CH, Cy), 33.3 (CH_2, Cy),

25.0 (CH_2, Cy), 24.6 (CH_2, Cy) ppm. These NMR data matched those reported in the literature.[1]

NMR spectra:

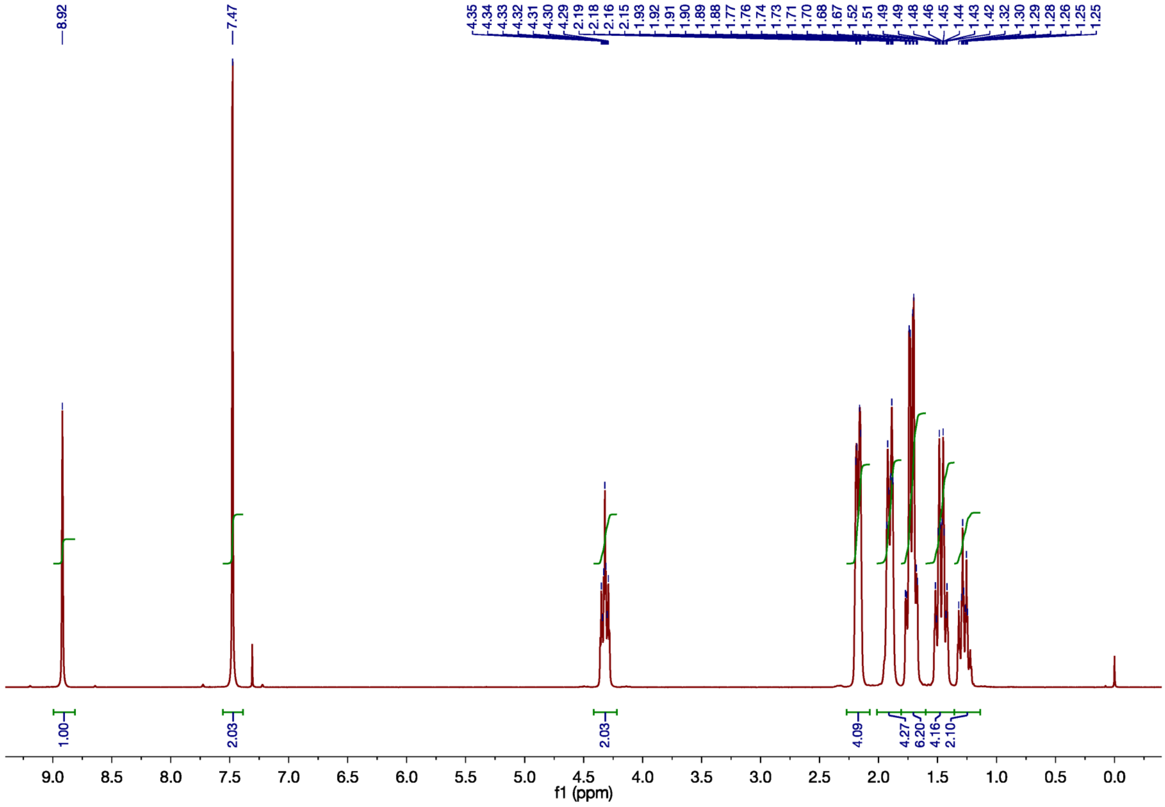

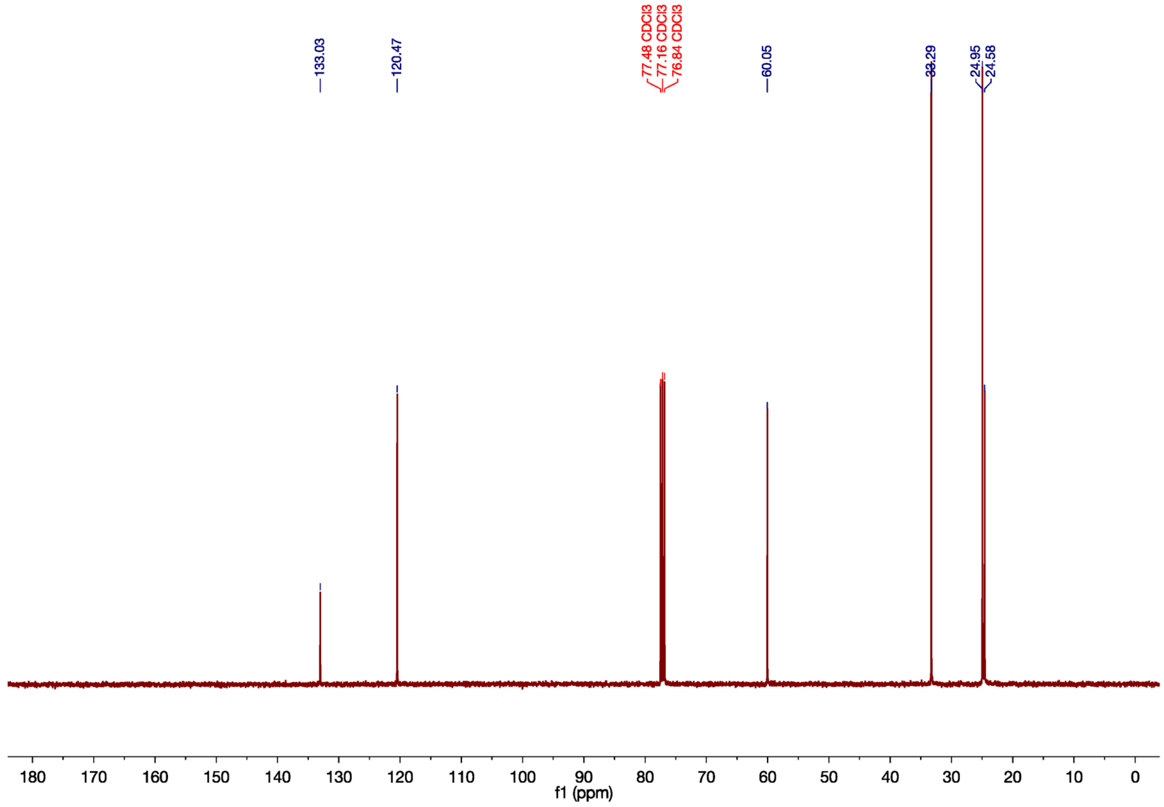

3. Synthesis of 1,3-dibenzylimidazolium tetrafluoroborate (IBn·HBF_4)

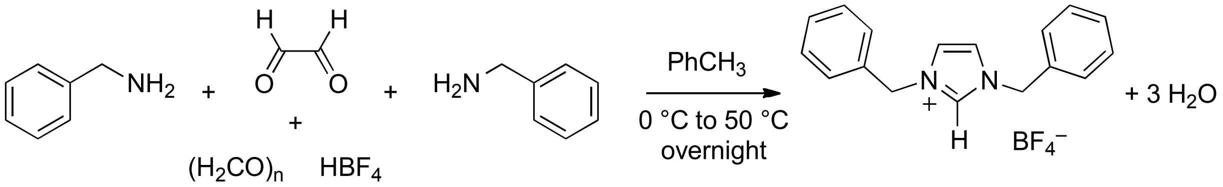

MF	C_7H_9N	$(CH_2O)_n$	$C_2H_2O_2$	HBF_4	$C_{17}H_{17}BF_4N_2$
MW	107.1548	30.0262	58.0366	87.8125	336.1393
amount (mmol)	2 × 100	100	100	125	100
mass (g)	2 × 10.7155	3.0026	14.51 g of 40% aq. sln	22.87 g of 48% aq. sln	33.6139 (100% yield)

Experimental procedure: A 500 mL round-bottom flask equipped with a magnetic stirring bar and a stopper was charged with benzylamine (10.72 g, 100 mmol), toluene (100 mL), and paraformaldehyde (3.00 g, 100 mmol). The resulting milky suspension was stirred for 30 min at room temperature until the solid was almost completely dissolved. The mixture was cooled to 0 °C in an ice/water bath and a second equivalent of benzylamine (10.72 g, 100 mmol) was added. The resulting solution was stirred for 10 min at 0 °C before tetrafluoroboric acid (48% aqueous solution, 22.87 g, 16.3 mL, 125 mmol) was added portionwise in 15 min. The cooling bath was removed and glyoxal (40 % aqueous solution, 14.51 g, 11.5 mL, 100 mmol) was added. The resulting cloudy mixture was stirred overnight (ca. 12 h) in an oil bath at 50 °C. After cooling to room temperature, dichloromethane (100 mL) and water (50 mL) were added and the layers were separated. The aqueous phase was extracted with 3 portions of dichloromethane (100 mL each). The organic layers were combined and dried over $MgSO_4$. After evaporation of the solvent, the yellow-beige residue was recrystallized from isopropanol to afford pure 1,3-dibenzylimidazolium tetrafluoroborate as large chunks of a pale brown solid (typical yield: 27 g, 80%).

Analytical data: Mp 89–91 °C (lit. 87–90 °C).[2] ^{1}H NMR (400 MHz, $CDCl_3$): δ = 8.95 (s, 1 H, CH^2 Im), 7.31–7.34 (m, 4 H, *m*-CH Ph), 7.24–7.27 (m, 6 H, *o*-CH + *p*-CH Ph), 7.17 (d, $^3J_{H,H}$ = 4.0 Hz, 2 H, $CH^{4,5}$ Im), 5.21 (s, 4 H, CH_2) ppm. ^{13}C NMR (100 MHz, $CDCl_3$): δ =

135.5 (CH^2 Im), 132.9 (*i*-C Ph), 129.43 (*p*-CH Ph), 129.39 (*m*-CH Ph), 128.9 (*o*-CH Ph), 122.4 (CH^{4,5} Im), 53.3 (CH_2) ppm.

NMR spectra:

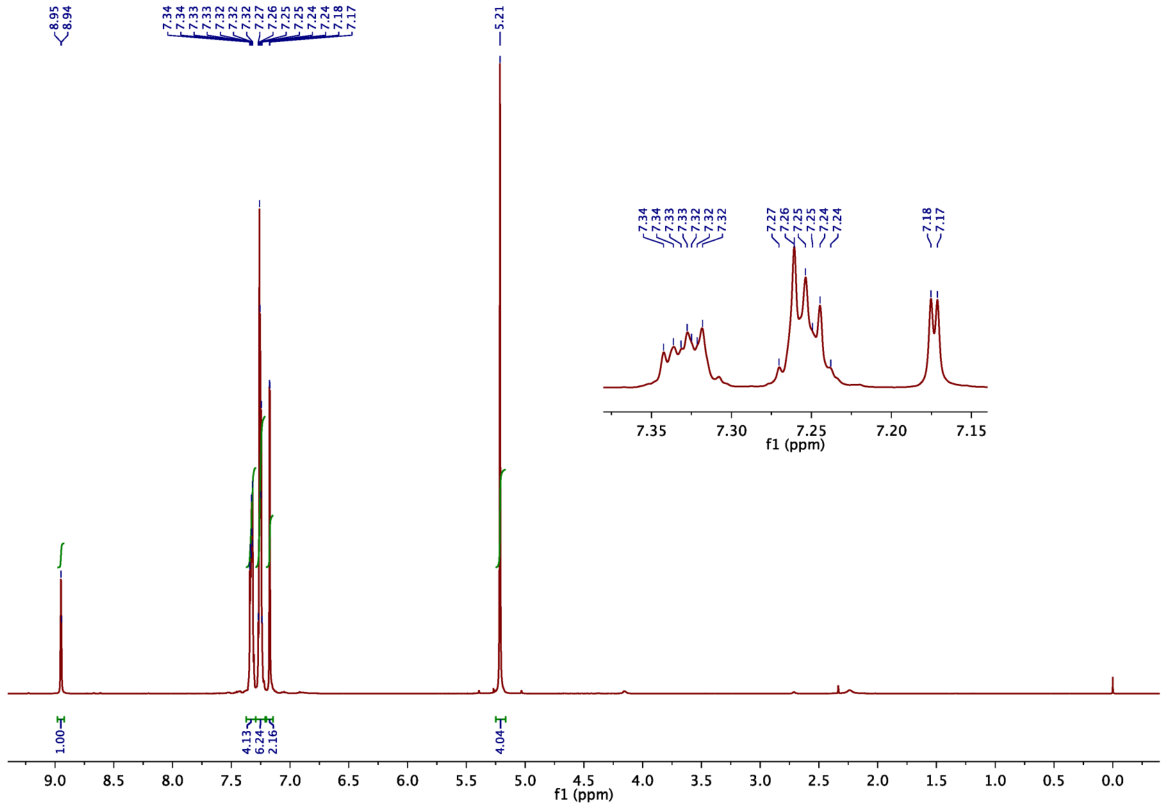

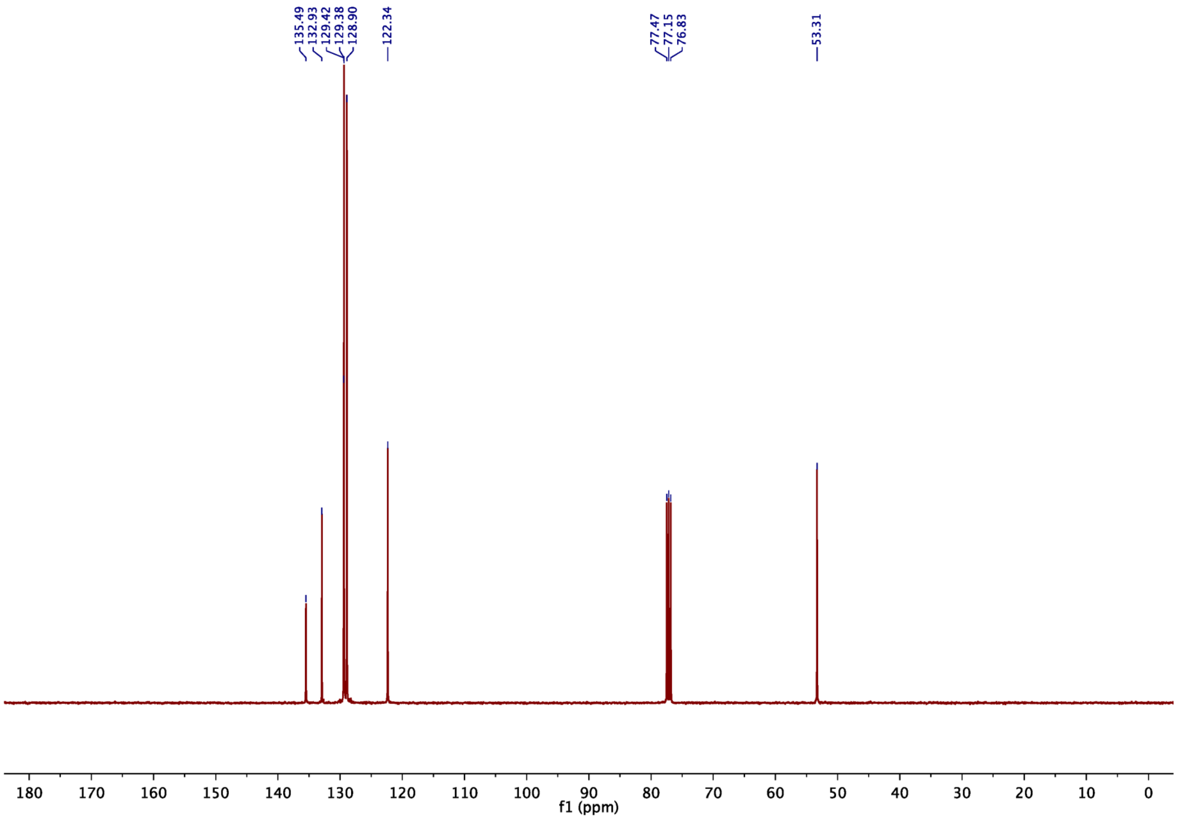

4. Synthesis of *N,N′*-dimesitylethylenediimine

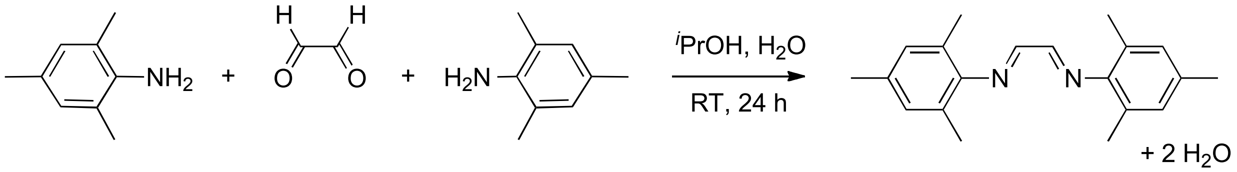

MF	$C_9H_{13}N$	$C_2H_2O_2$	$C_{20}H_{24}N_2$
MW	135.2084	58.0366	292.4230
amount (mmol)	300	150	150
mass (g)	40.5625	21.7637 g of 40% aqueous solution	43.8635 (100% yield)

Experimental procedure: A 500 mL round-bottom flask equipped with a magnetic stirring bar and a stopper was charged with 2,4,6-trimethylaniline (40.56 g, 300 mmol) and isopropanol (150 mL). In a 250 mL Erlenmeyer flask, glyoxal (40 % aqueous solution, 21.76 g, 17.3 mL, 150 mmol) was diluted with water (50 mL) and isopropanol (50 mL). The colorless solution of glyoxal was added to the orange-brown mesitylamine solution in one portion. The resulting yellow-orange solution was stirred for 24 h at room temperature. A bright yellow precipitate appeared within a few minutes. After 24 h, the yellow-brown suspension was filtered through a Büchner funnel and the precipitate was rinsed twice with water (2 × 100 mL). It was spread in an evaporating dish and dried until constant weight under an IR lamp to give a bright yellow powder that was pure enough for the next step (typical yield: >39.5 g, >90% yield).[3]

Analytical data: Mp 157.1–157.3 °C (lit. 157 °C).[4] ^{1}H NMR (400 MHz, $CDCl_3$): δ = 8.10 (s, 2 H, CH=N), 6.90 (s, 4 H, *m*-CH), 2.29 (s, 6 H, *p*-CH_3), 2.16 (s, 12 H, *o*-CH_3) ppm. ^{13}C NMR (100 MHz, $CDCl_3$): δ = 163.6 (CH=N), 147.4 (*i*-C Mes), 134.4 (*p*-C Mes), 129.0 (*m*-CH Mes), 126.6 (*o*-C Mes), 20.9 (*p*-CH_3), 18.4 (*o*-CH_3) ppm.

NMR spectra:

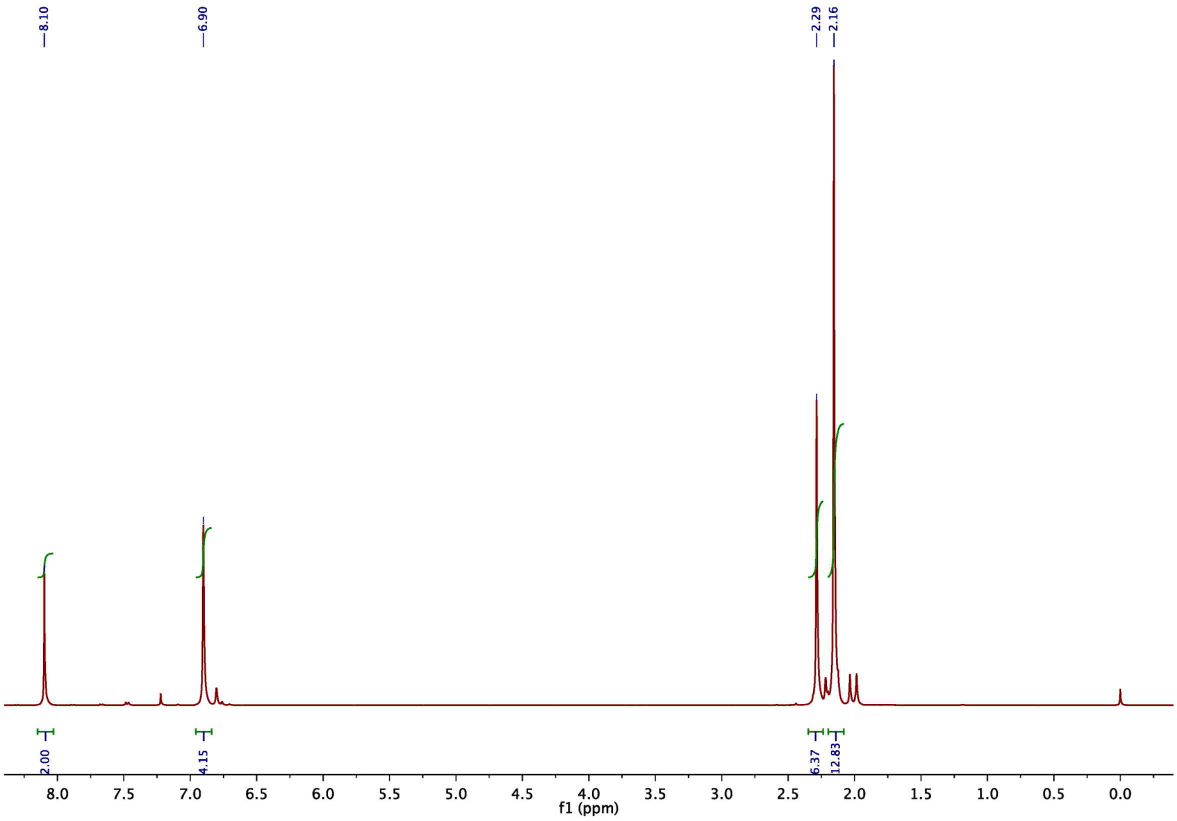

8.10
6.90
2.29
2.16
2.00
4.15
6.37
12.83
8.0
7.5
7.0
6.5
6.0
5.5
5.0
4.5
4.0
3.5
3.0
2.5
2.0
1.5
1.0
0.5
0.0
f1 (ppm)

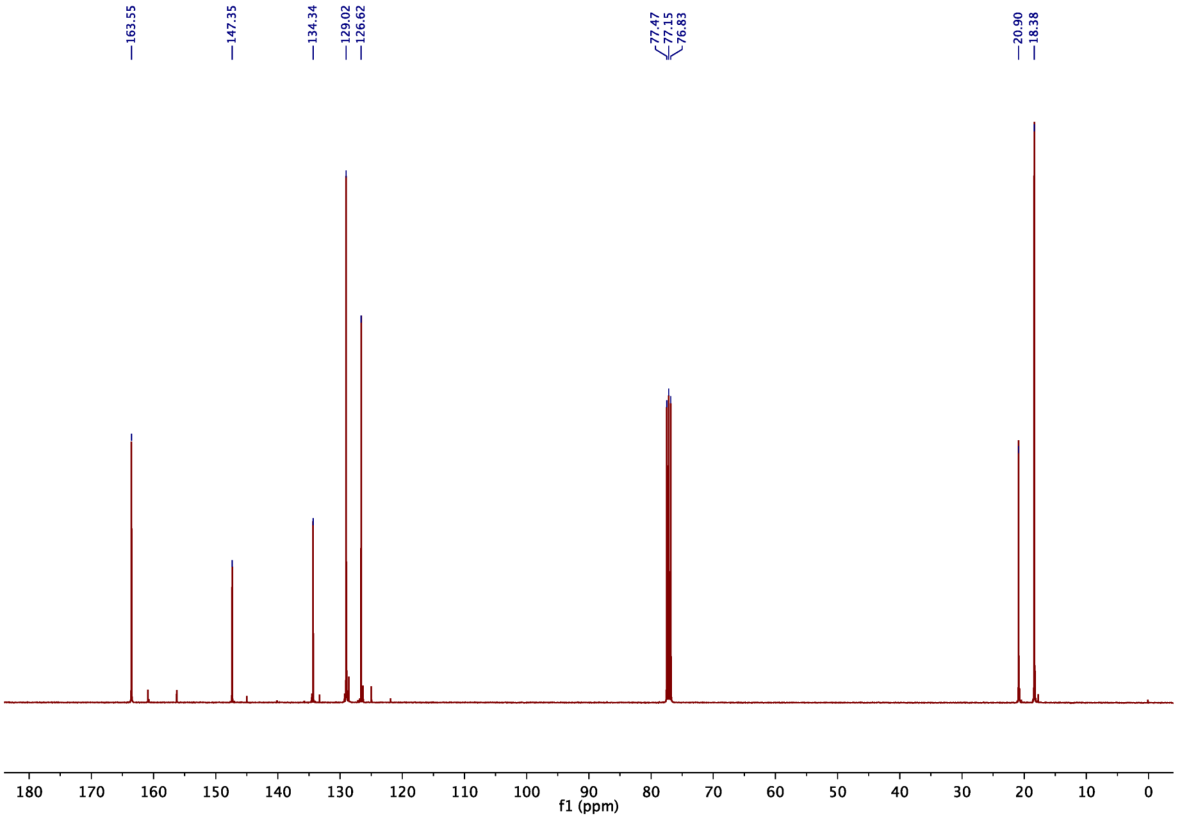

163.55
147.35
134.34
129.02
126.62
77.47
77.15
76.83
20.90
18.38
180
170
160
150
140
130
120
110
100
90
80
70
60
50
40
30
20
10
0
f1 (ppm)

5. Synthesis of 1,3-dimesitylimidazolium chloride (IMes·HCl)

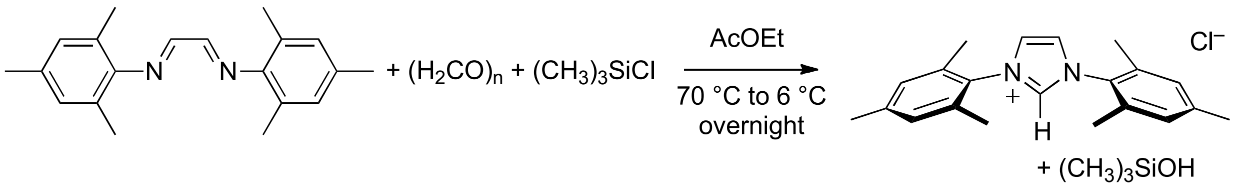

MF	$C_{20}H_{24}N_2$	$(CH_2O)_n$	C_3H_9SiCl	$C_{21}H_{25}ClN_2$
MW	292.4230	30.0262	108.6426	340.8949
amount (mmol)	200	200	200	200
mass (g)	58.4846	6.0052	21.7285	68.1790 (100% yield)

Experimental procedure: A 1 L round-bottom flask equipped with a magnetic stirring bar and a stopper was charged with ethyl acetate (800 mL) and heated in an oil bath at 70 °C. To the warm solvent were added *N,N'*-dimesitylethylenediimine (58.48 g, 200 mmol) and paraformaldehyde (6.00 g, 200 mmol). Next, a solution of chlorotrimethylsilane (21.73 g, 25.4 mL, 200 mmol) in ethyl acetate (25 mL) was added dropwise over 45 min with vigorous stirring. The resulting yellow-orange suspension was further stirred for 2 h at 70 °C. The color changed to brown-black and the mixture was slowly cooled to room temperature. It was kept overnight in a cold room at +6 °C. The cold suspension was filtered through a Büchner funnel and the white precipitate was rinsed twice with ethyl acetate (2 × 100 mL) and once with diethylether (100 mL). It was spread in an evaporating dish and dried until constant weight under an IR lamp to afford pure 1,3-dimesitylimidazolium chloride as a white micro-crystalline powder (typical yield: 58 g, 85% yield).

Analytical data: Mp 335–337 °C (dec.). ^{1}H NMR (400 MHz, $CDCl_3$): δ = 10.48 (s, 1 H, CH^2 Im), 7.76 (s, 2 H, $CH^{4,5}$ Im), 6.99 (s, 4 H, *m*-CH Mes), 2.32 (s, 6 H, *p*-CH_3 Mes), 2.13 (s, 12 H, *o*-CH_3 Mes) ppm. ^{13}C NMR (100 MHz, $CDCl_3$): δ = 141.0 (*p*-C Mes), 138.8 (CH^2 Im), 134.0 (*o*-C Mes), 130.6 (*i*-C Mes), 129.7 (*m*-CH Mes), 124.9 ($CH^{4,5}$ Im), 21.0 (*p*-CH_3), 17.5 (*o*-CH_3) ppm. These NMR data matched those reported in the literature.[5]

NMR spectra:

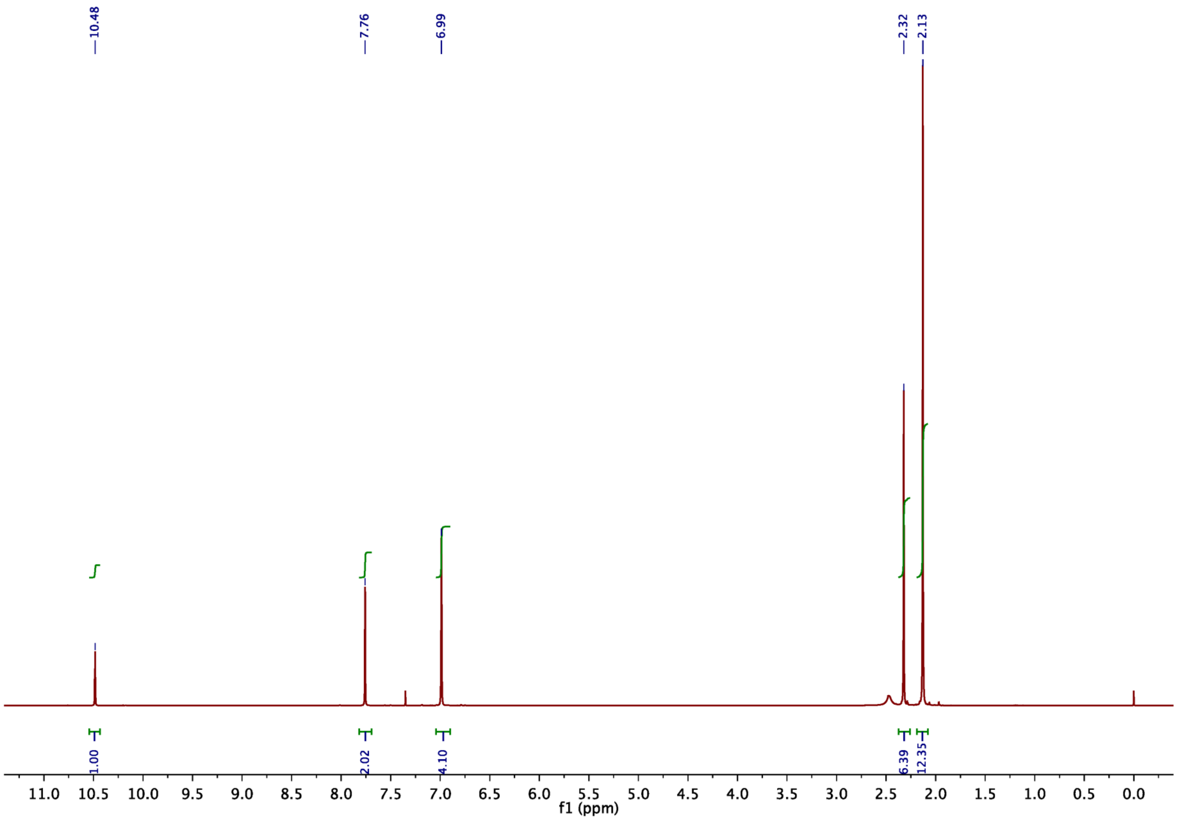
10.48
7.76
6.99
2.32
2.13
1.00
2.02
4.10
6.39
12.35
11.0 10.5 10.0 9.5 9.0 8.5 8.0 7.5 7.0 6.5 6.0 5.5 5.0 4.5 4.0 3.5 3.0 2.5 2.0 1.5 1.0 0.5 0.0
f1 (ppm)

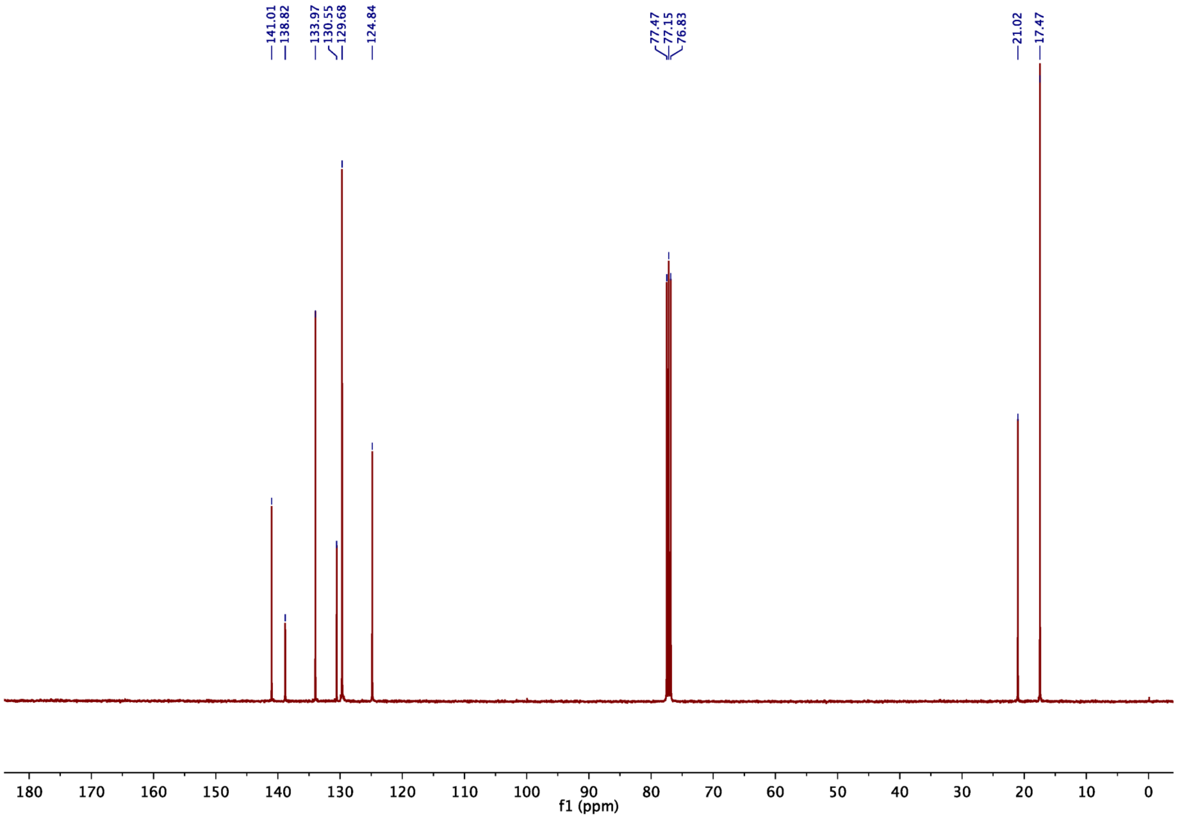
141.01
138.82
133.97
130.55
129.68
124.84
77.47
77.15
76.83
21.02
17.47
180 170 160 150 140 130 120 110 100 90 80 70 60 50 40 30 20 10 0
f1 (ppm)

6. Synthesis of 1,3-dimesitylimidazolium tetrafluoroborate (IMes·HBF_4)

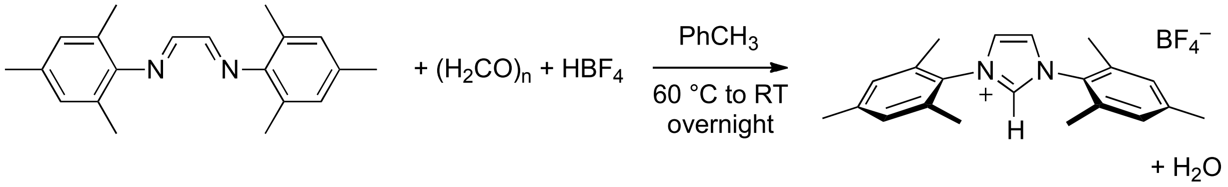

MF	$C_{20}H_{24}N_2$	$(CH_2O)_n$	HBF_4	$C_{21}H_{25}BF_4N_2$
MW	292.4230	30.0262	87.8125	392.2465
amount (mmol)	50	60	70	50
mass (g)	14.6212	1.8016	12.8060 g of 48% aqueous solution	19.6123 (100% yield)

Experimental procedure: A 500 mL round-bottom flask equipped with a magnetic stirring bar and a stopper was charged with paraformaldehyde (1.80 g, 60 mmol), toluene (200 mL) and tetrafluoroboric acid (48% aqueous solution, 12.81 g, 9.1 mL, 70 mmol). The mixture was stirred until complete dissolution of the paraformaldehyde. Next, *N,N'*-dimesitylethylene-diimine (14.62 g, 50 mmol) was added in small portions over a 10 min period of time. The resulting brown suspension was stirred for 4 h in an oil bath at 60 °C to afford a black slurry, which was slowly cooled down and further stirred overnight at room temperature. The precipitate was filtered through a Büchner funnel, washed with ethyl acetate (4 × 10 mL), and dried under high vacuum. The brown-grey crude product was recrystallized from isopropanol to afford pure 1,3-dimesitylimidazolium tetrafluoroborate as a white microcrystalline powder (typical yield: 15.69 g, 80%).

Analytical data: Mp 255–257 °C. ^1H NMR (400 MHz, $CDCl_3$): δ = 8.82 (s, 1 H, CH^2 Im), 7.55 (s, 2 H, $CH^{4,5}$ Im), 6.98 (s, 4 H, *m*-CH Mes), 2.31 (s, 6 H, *p*-CH_3 Mes), 2.06 (s, 12 H, *o*-CH_3 Mes) ppm. ^{13}C NMR (100 MHz, $CDCl_3$): δ = 141.1 (*p*-C Mes), 137.0 (CH^2 Im), 134.0 (*o*-C Mes), 130.4 (*i*-C Mes), 129.7 (*m*-CH Mes), 125.2 ($CH^{4,5}$ Im), 21.1 (*p*-CH_3), 17.1 (*o*-CH_3) ppm. These data matched those reported in the literature.[6]

NMR spectra:

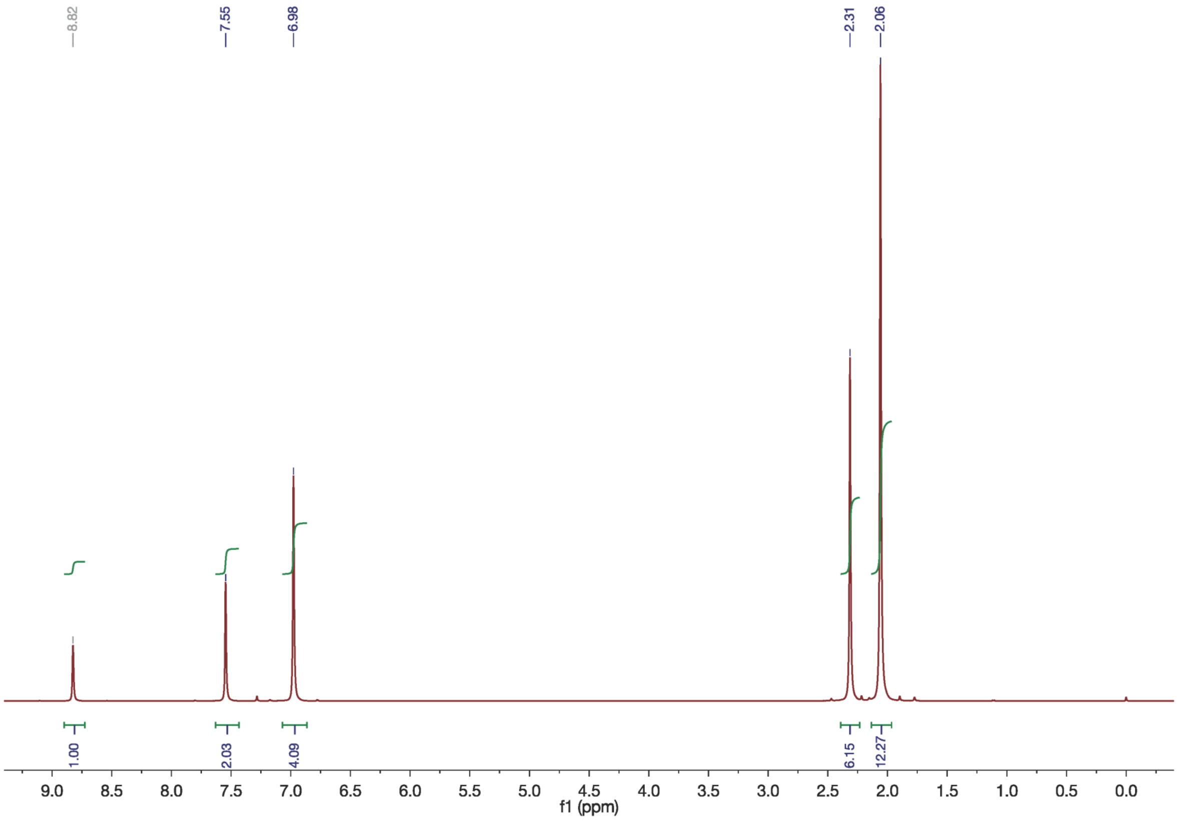
8.82
7.55
6.98
2.31
2.06
1.00
2.03
4.09
6.15
12.27
9.0 8.5 8.0 7.5 7.0 6.5 6.0 5.5 5.0 4.5 4.0 3.5 3.0 2.5 2.0 1.5 1.0 0.5 0.0
f1 (ppm)

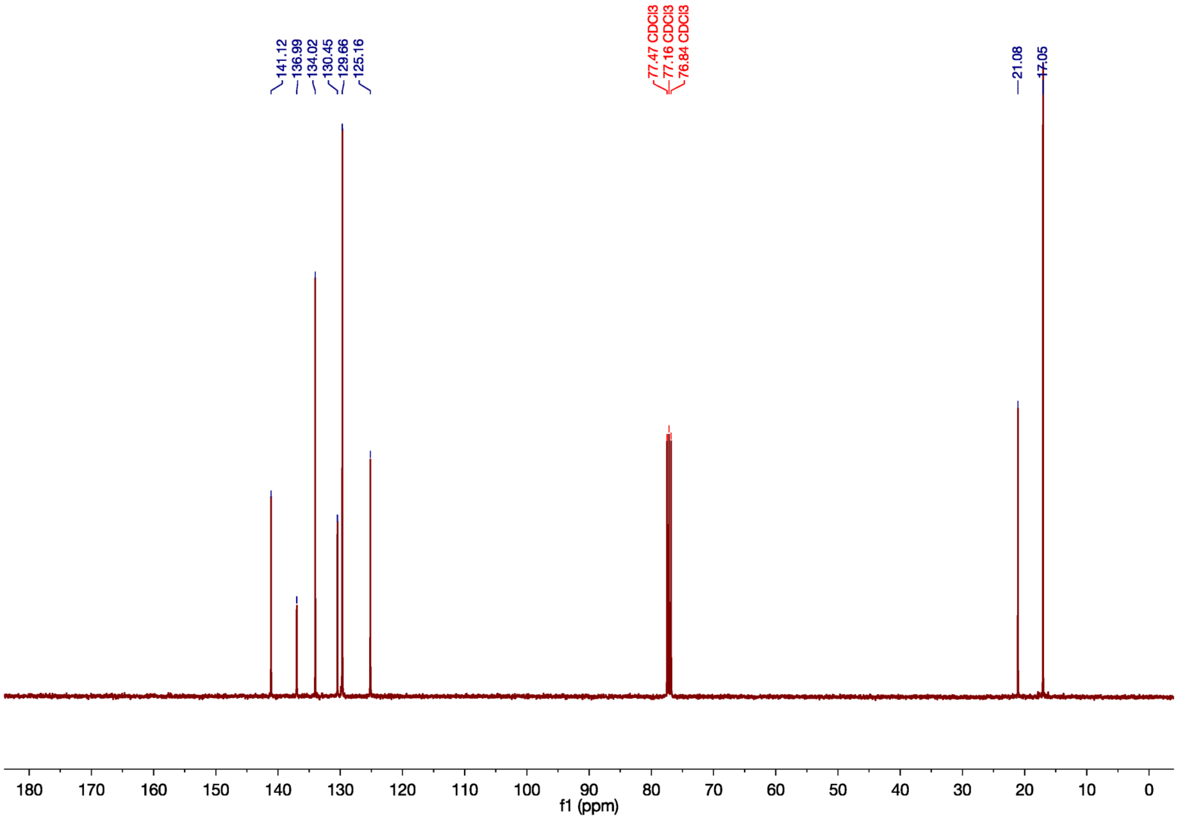
141.12
136.99
134.02
130.45
129.66
125.16
77.47 CDCl3
77.16 CDCl3
76.84 CDCl3
21.08
17.05
180 170 160 150 140 130 120 110 100 90 80 70 60 50 40 30 20 10 0
f1 (ppm)

7. Synthesis of *N,N'*-dimesitylethylenediamine dihydrochloride

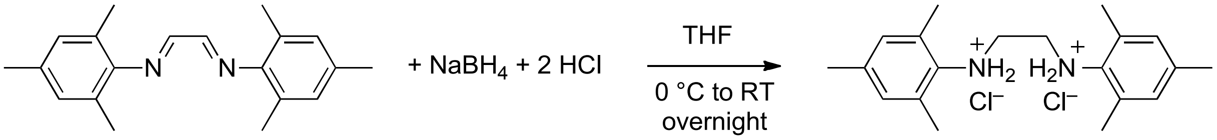

MF	$C_{20}H_{24}N_2$	$NaBH_4$	HCl	$C_{21}H_{25}BF_4N_2$
MW	292.4230	37.8324	36.4609	369.3764
amount (mmol)	60	240	120	60
mass (g)	17.5454	9.0798	12.1536 g of 36% aqueous solution	22.1626 (100% yield)

Experimental procedure: A 1 L beaker equipped with a magnetic stirring bar was charged with *N,N'*-dimesitylethylenediimine (17.54 g, 60 mmol) and THF (300 mL). The resulting yellow suspension was cooled to 0 °C in an ice/water bath before sodium borohydride (9.08 g, 240 mmol) was added in one portion. Next, concentrated hydrochloric acid (12.15 g, 10.1 mL, 120 mmol) was added dropwise in 30 min and the reaction mixture was further stirred at 0 °C for 1 h. Cold 3 M aqueous hydrochloric acid prepared by diluting 120 mL of concentrated hydrochloric acid into 360 g of ice (480 mL, 1.44 mol) was added dropwise to the resulting white suspension maintained at 0 °C in an ice/water bath. The resulting yellow suspension was warmed up slowly to room temperature and further stirred overnight. The white precipitate was filtered through a Büchner funnel and rinsed with water (3 × 100 mL). It was spread in an evaporating dish and dried until constant weight under an IR lamp to give a white powder that was pure enough for the next step (typical yield: 16.6 g, 75% yield).

Analytical data: Mp 224–225 °C (dec.). ^{1}H NMR (400 MHz, DMSO-d_6): δ = 6.95 (s, 4 H, *m*-CH), 3.58 (s, 4 H, CH_2N), 2.43 (s, 12 H, *o*-CH_3), 2.22 (s, 6 H, *p*-CH_3) ppm. ^{13}C NMR (100 MHz, DMSO-d_6): δ = 136.3 (C_{ar} Mes), 134.3 (C_{ar} Mes), 131.4 (C_{ar} Mes), 130.2 (*m*-CH Mes), 46.6 (CH_2N), 20.3 (*p*-CH_3), 18.2 (*o*-CH_3) ppm. These data matched those reported in the literature.[4]

NMR spectra:

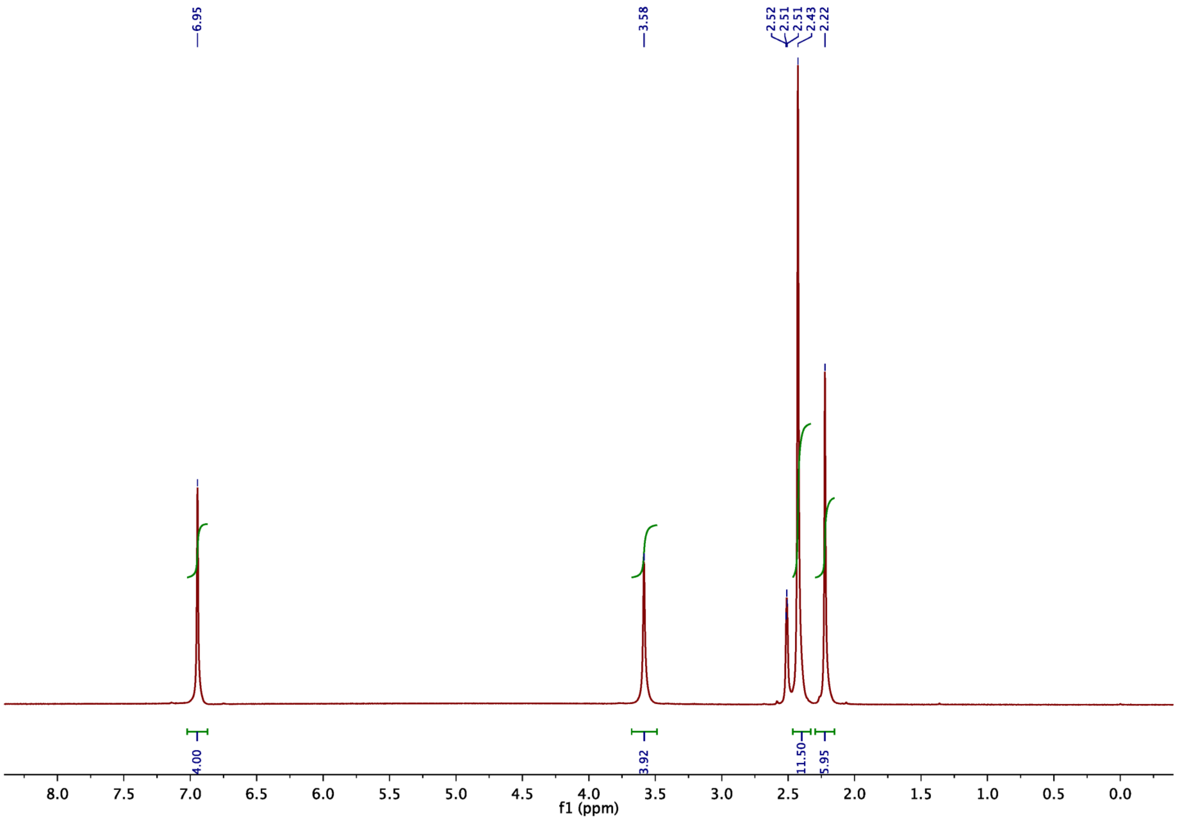
6.95
3.58
2.52
2.51
2.51
2.43
2.22
4.00
3.92
11.50
5.95
8.0 7.5 7.0 6.5 6.0 5.5 5.0 4.5 4.0 3.5 3.0 2.5 2.0 1.5 1.0 0.5 0.0
f1 (ppm)

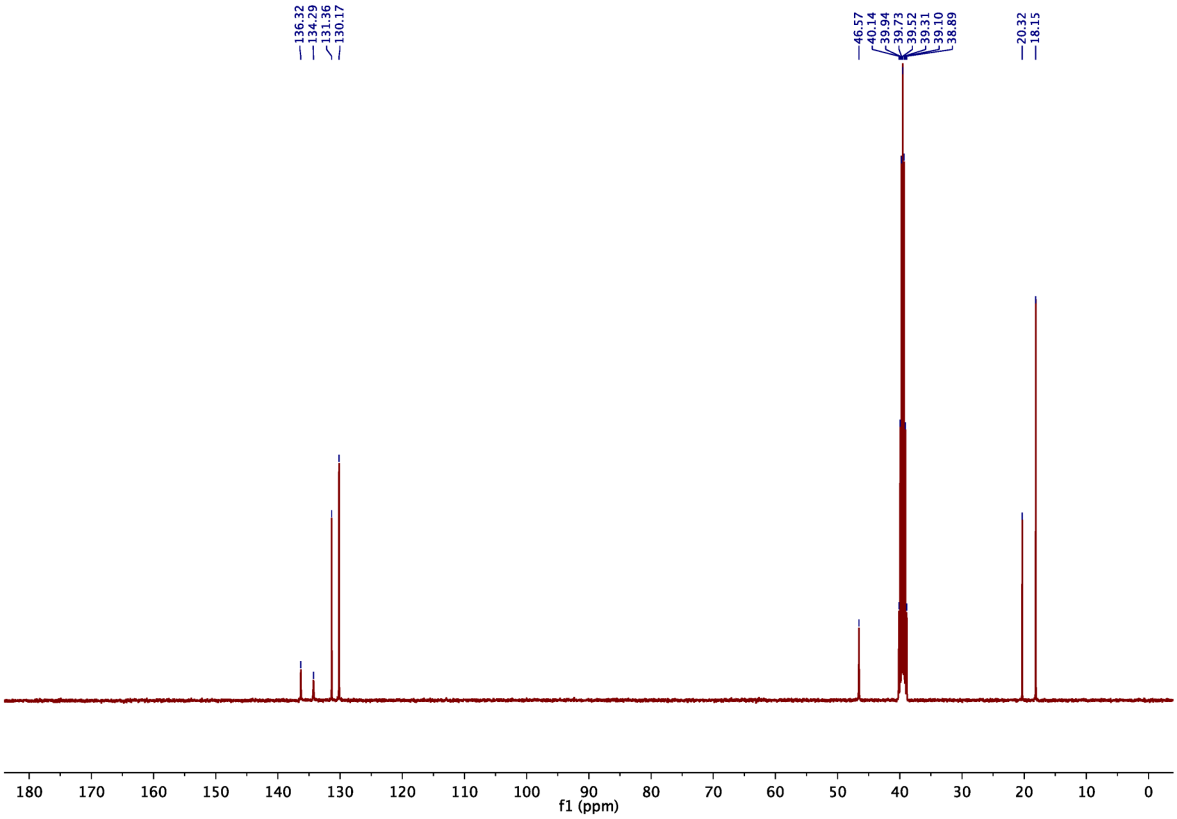
136.32
134.29
131.36
130.17
46.57
40.14
39.94
39.73
39.52
39.31
39.10
38.89
20.32
18.15
180 170 160 150 140 130 120 110 100 90 80 70 60 50 40 30 20 10 0
f1 (ppm)

8. Synthesis of 1,3-dimesitylimidazolinium chloride (SIMes·HCl)

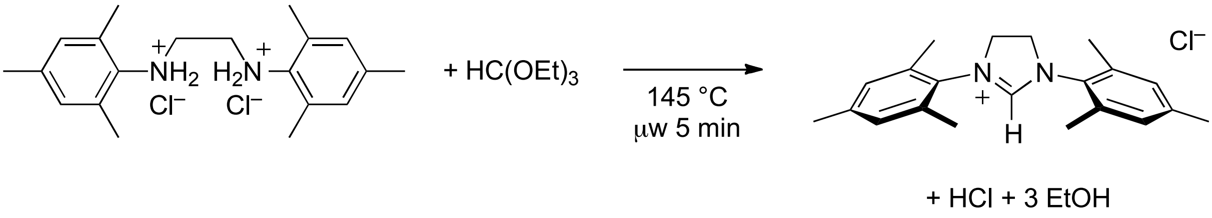

MF	$C_{20}H_{30}Cl_2N_2$	$C_7H_{16}O_3$	$C_{21}H_{27}ClN_2$
MW	369.3764	148.2016	342.9107
amount (mmol)	50	150	50
mass (g)	18.4688	22.2302	17.1455 (100% yield)

Experimental procedure: A 80 mL pressure vial equipped with a magnetic stirring bar was charged with *N,N'*-dimesitylethylenediamine dihydrochloride (18.47 g, 50 mmol) and triethyl orthoformate (22.23 g, 25 mL, 150 mmol). The mixture was triturated with a spatula until a homogeneous thick white paste was obtained. The vial was closed with a screw cap equipped with a pressure sensor and a glass inlet tube ended by a sapphire window containing an optical fiber temperature sensor. The reaction mixture was heated for 5 min at 145 °C in a monomodal microwave reactor. The maximum microwave power was set to 150 W and the pressure limit at 250 psi. No ramp and no simultaneous cooling were applied. After rapid air cooling by the unit, the resulting beige-yellow suspension was poured into a 250 mL beaker placed in an ice bath and the reaction vessel was rinsed twice with diethyl ether (2 × 50 mL). The beige suspension was filtered on a Büchner funnel and the off-white precipitate was rinsed with diethyl ether (2 × 50 mL). The crude product was dissolved in hot acetonitrile (50 mL), briefly cooled to room temperature, followed by addition of diethyl ether (200 mL). The pale yellow suspension was filtered on a Büchner funnel and the white precipitate was washed with diethyl ether (50 mL). It was spread in an evaporating dish and dried until constant weight under an IR lamp to afford pure 1,3-dimesitylimidazolinium chloride as a white micro-crystalline powder (typical yield: 12 g, 70% yield).

Analytical data: Mp 279–280 °C (lit. 279–281 °C).[4] ^{1}H NMR (400 MHz, $CDCl_3$): $\delta = 9.62$ (s, 1 H, CH^2 Im), 6.88 (s, 4 H *m*-CH Mes), 4.50 (s, 4 H, $CH_2^{4,5}$ Im), 2.33 (s, 12 H, *o*-CH_3),

2.25 (s, 6 H, *p*-CH_3) ppm. ^{13}C NMR (100 MHz, $CDCl_3$): δ = 160.0 (CH^2 Im), 140.2 (*p*-C Mes), 135.0 (*o*-C Mes), 130.3 (*i*-C Mes), 129.8 (*m*-CH Mes), 51.9 ($CH_2^{4,5}$ Im), 21.0 (*p*-CH_3), 17.9 (*o*-CH_3) ppm.

NMR spectra:

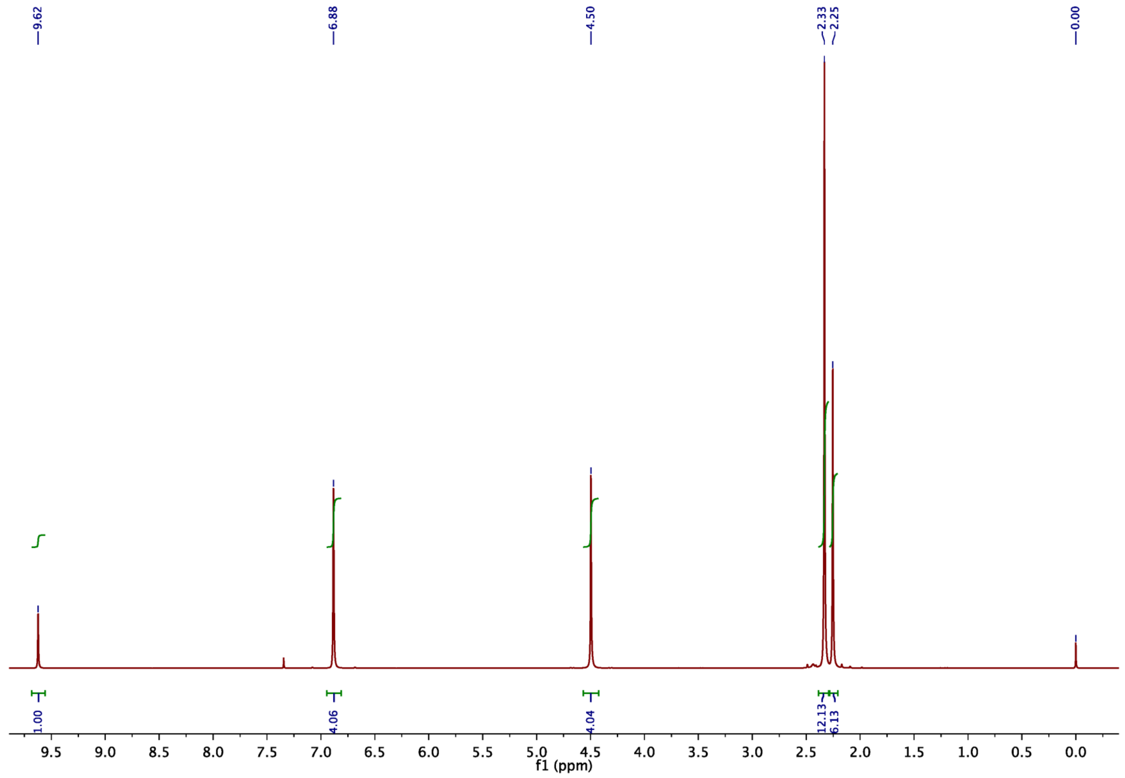

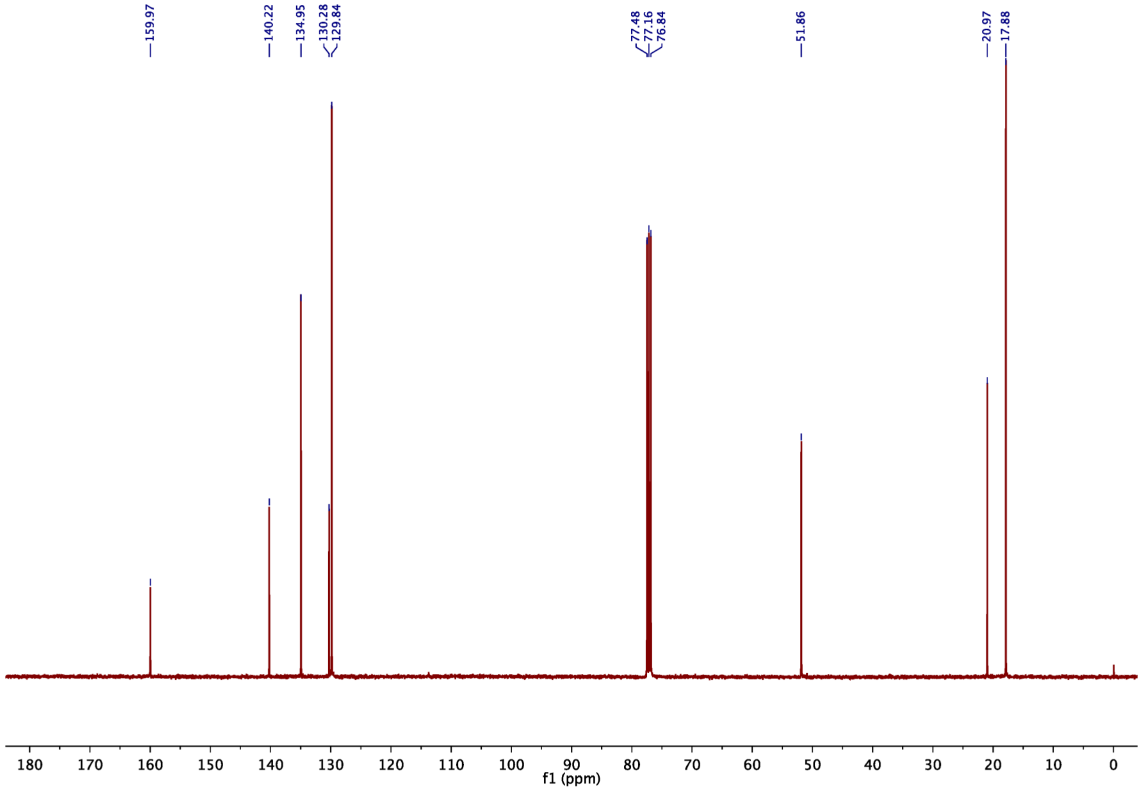

9. Synthesis of *N,N'*-bis(2,6-diisopropylphenyl)ethylenediimine

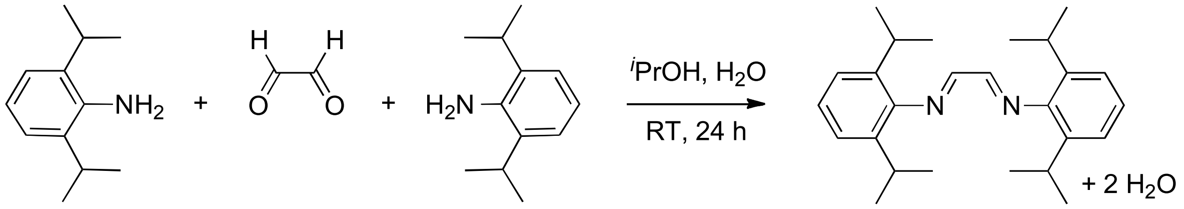

MF	$C_{12}H_{19}N$	$C_2H_2O_2$	$C_{26}H_{36}N_2$
MW	177.2888	58.0366	376.5838
amount (mmol)	300	150	150
mass (g)	59.0963 g of 90% techn. grade reagent	21.7637 g of 40% aqueous solution	56.4876 (100% yield)

Experimental procedure: A 500 mL round-bottom flask equipped with a magnetic stirring bar and a stopper was charged with 2,6-diisopropylaniline (59.10 g of 90% technical grade reagent, 300 mmol) and isopropanol (150 mL). In a 250 mL Erlenmeyer flask, glyoxal (40 % aqueous solution, 21.76 g, 17.3 mL, 150 mmol) was diluted with water (50 mL) and isopropanol (50 mL). The colorless solution of glyoxal was added to the beige-brown solution of 2,6-diisopropylaniline in one portion. The resulting yellow-brown solution was stirred for 24 h at room temperature. A yellow precipitate appeared within a few minutes. After 24 h, the yellow-brown suspension was filtered through a Büchner funnel and the precipitate was rinsed twice with water (2 × 100 mL). It was spread in an evaporating dish and dried until constant weight under an IR lamp to give a yellow powder that was pure enough for the next step (typical yield: >45.2 g, >80% yield).[3]

Analytical data: Mp 104.9–105.3 °C (lit. 105 °C).[7] ^{1}H NMR (400 MHz, $CDCl_3$): $\delta = 8.11$ (s, 2 H, CH=N), 7.16 (m, 6 H, CH_{ar}), 2.95 (sept, $^3J_{H,H} = 6.8$ Hz, 4 H, C*H*(CH_3)$_2$), 1.21 (d, $^3J_{H,H} =$ 6.8 Hz, 24 H, CH(C*H*$_3$)$_2$) ppm. ^{13}C NMR (100 MHz, $CDCl_3$): $\delta = 163.2$ (CH=N), 148.1 (*i*-C Dip), 136.8 (*o*-C Dip), 125.3 (*p*-CH Dip), 123.3 (*m*-CH Dip), 28.2 (*C*H(CH_3)$_2$), 23.5 (CH(*C*H$_3$)$_2$) ppm.

NMR spectra:

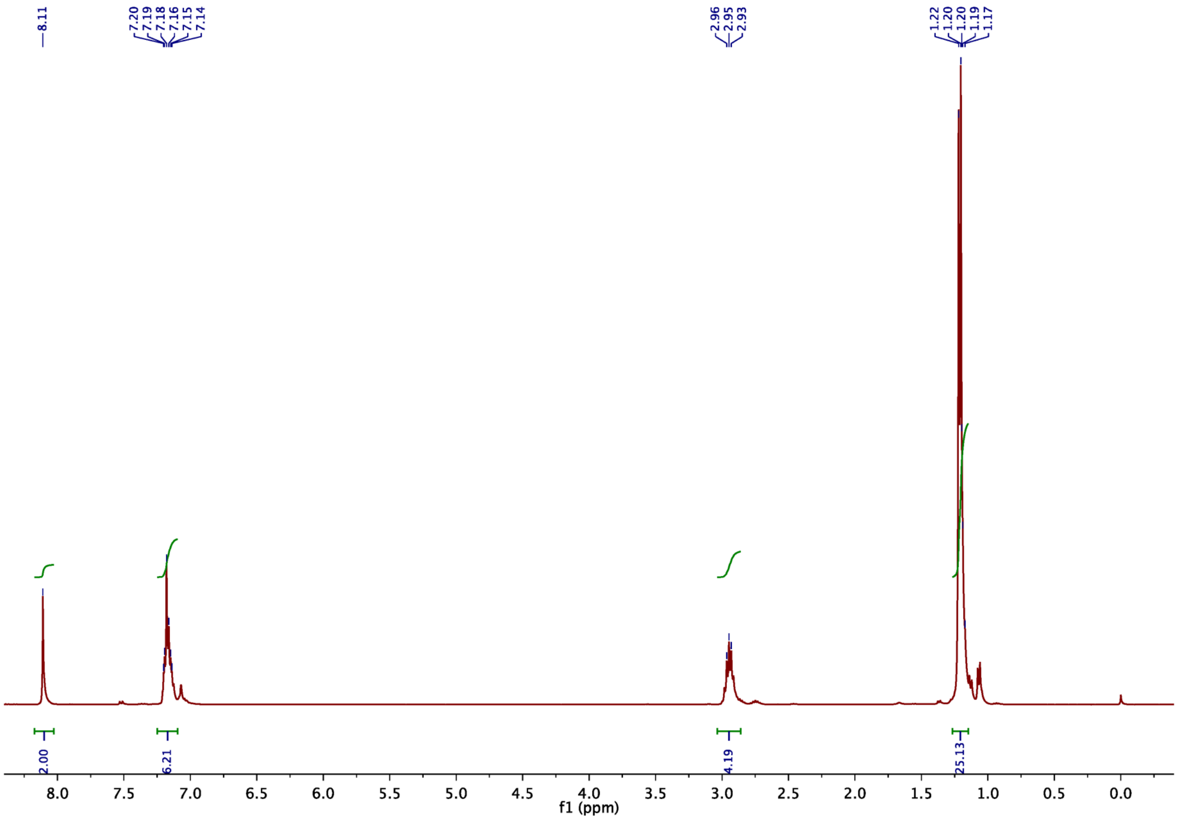

8.11
7.20
7.19
7.18
7.16
7.15
7.14
2.96
2.95
2.93
1.22
1.20
1.20
1.19
1.17
2.00
6.21
4.19
25.13
f1 (ppm)

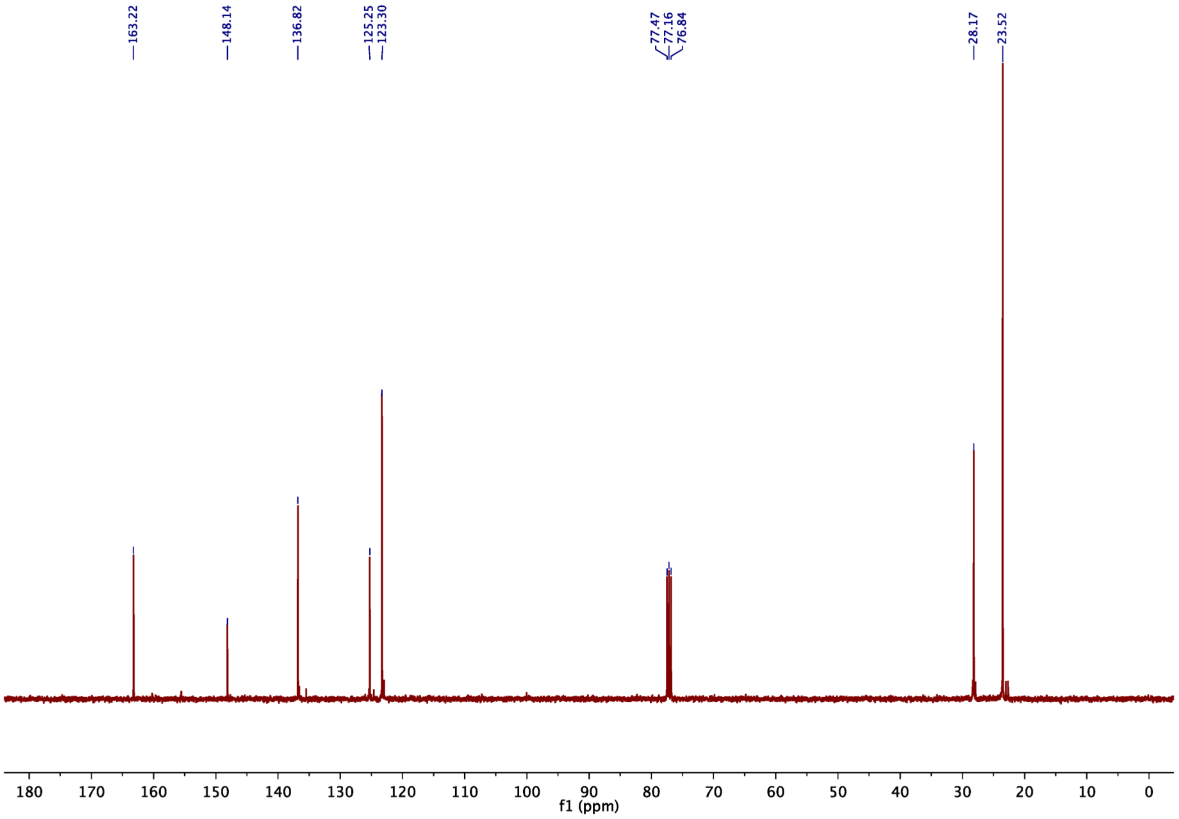

163.22
148.14
136.82
125.25
123.30
77.47
77.16
76.84
28.17
23.52
f1 (ppm)

10. Synthesis of 1,3-bis(2,6-diisopropylphenyl)imidazolium chloride (IDip·HCl)

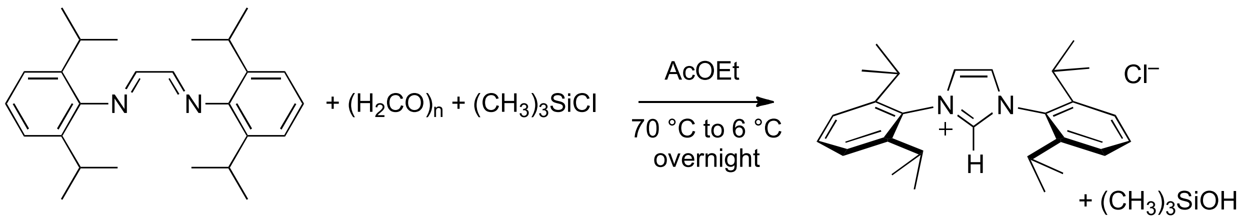

MF	$C_{26}H_{36}N_2$	$(CH_2O)_n$	C_3H_9SiCl	$C_{27}H_{37}ClN_2$
MW	376.5838	30.0262	108.6426	425.0557
amount (mmol)	100	100	100	100
mass (g)	37.6584	3.0026	10.8643	42.5056 (100% yield)

A 1 L round-bottom flask equipped with a magnetic stirring bar and a stopper was charged with ethyl acetate (800 mL) and heated in an oil bath at 70 °C. To the warm solvent were added *N,N'*-bis(2,6-diisopropylphenyl)ethylenediimine (37.66 g, 100 mmol) and paraformaldehyde (3.00 g, 100 mmol). Next, a solution of chlorotrimethylsilane (10.86 g, 12.7 mL, 100 mmol) in ethyl acetate (10 mL) was added dropwise over 45 min with vigorous stirring. The resulting yellow-orange suspension was further stirred for 2 h at 70 °C. The color changed to black-brown and the mixture was slowly cooled to room temperature. It was kept overnight in a cold room at +6 °C. The cold suspension was filtered through a Büchner funnel and the white precipitate was rinsed twice with ethyl acetate (2 × 100 mL) and once with diethylether (100 mL). It was spread in an evaporating dish and dried until constant weight under an IR lamp to afford pure 1,3-bis(2,6-diisopropylphenyl)imidazolium chloride as a white microcrystalline powder (typical yield: 36 g, 85% yield).

Analytical data: Mp 281–284 °C. ^{1}H NMR (400 MHz, DMSO-d_6): δ = 10.28 (s, 1 H, CH^2 Im), 8.60 (s, 2 H, CH^{4,5} Im), 7.69 (t, $^3J_{H,H}$ = 7.8 Hz, 2 H, *p*-CH Dip), 7.53 (d, $^3J_{H,H}$ = 8.0 Hz, 4 H, *m*-CH Dip), 2.36 (sept, $^3J_{H,H}$ = 6.8 Hz, 4 H, C*H*(CH$_3$)$_2$), 1.27 (d, $^3J_{H,H}$ = 6.8 Hz, 12 H, CH(C*H*$_3$)$_2$), 1.17 (d, $^3J_{H,H}$ = 6.8 Hz, 12 H, CH(C*H*$_3$)$_2$) ppm. ^{13}C NMR (100 MHz, DMSO-d_6): δ = 144.9 (*o*-C Dip), 139.4 (CH^2 Im), 131.9 (*p*-CH Dip), 130.1 (*i*-C Dip), 126.2 (CH^{4,5} Im),

124.7 (*m*-CH Dip), 28.7 (*C*H(CH_3)$_2$), 24.2 (CH(CH_3)$_2$), 23.2 (CH(CH_3)$_2$) ppm. These data matched those reported in the literature.[5]

NMR spectra:

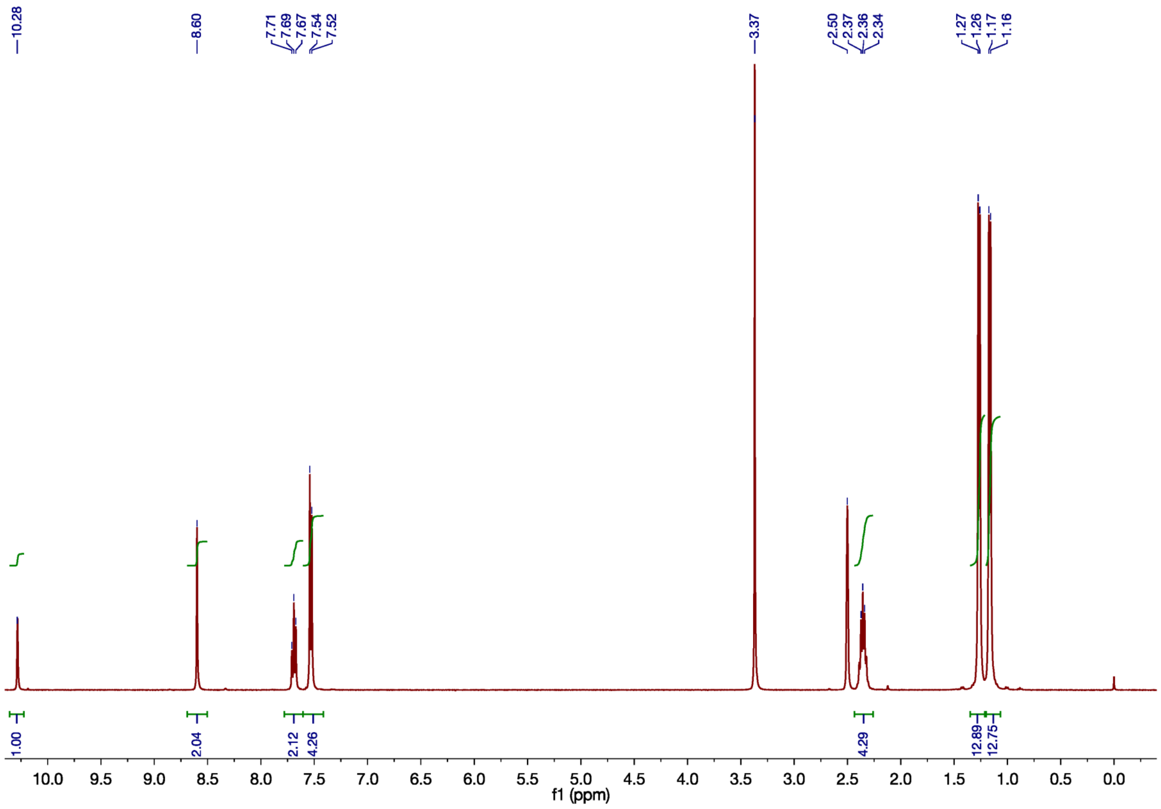

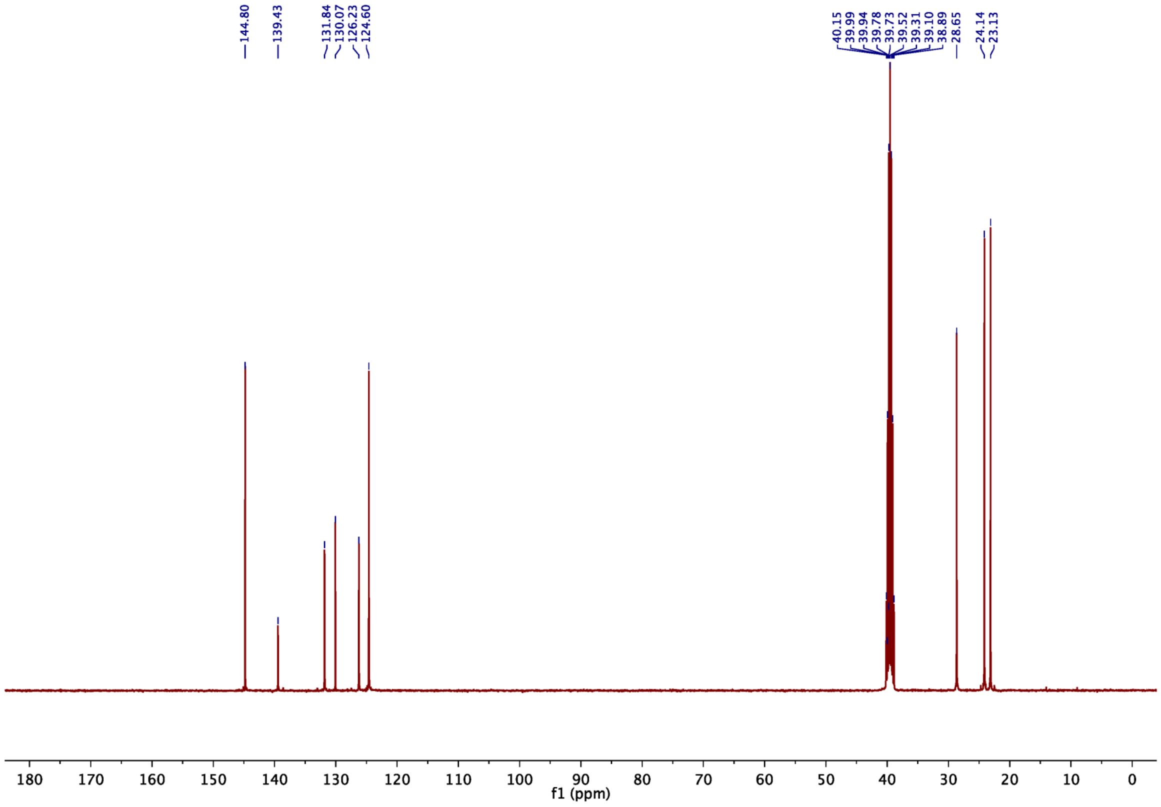

11. Synthesis of *N,N'*-bis(2,6-diisopropylphenyl)ethylenediamine dihydrochloride

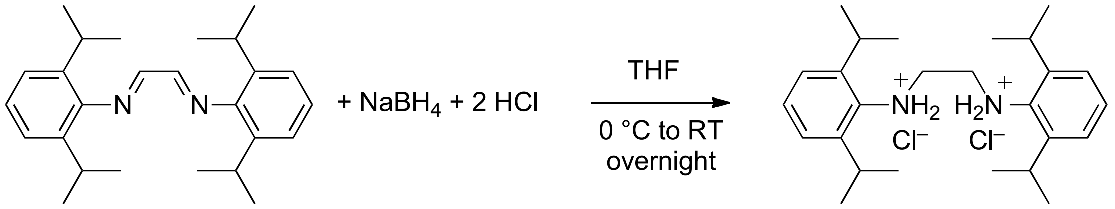

MF	$C_{26}H_{36}N_2$	$NaBH_4$	HCl	$C_{26}H_{42}Cl_2N_2$
MW	376.5838	37.8324	36.4609	453.5372
amount (mmol)	60	240	120	60
mass (g)	22.5950	9.0798	12.1536 g of 36% aqueous solution	27.2122 (100% yield)

Experimental procedure: A 1 L beaker equipped with a magnetic stirring bar was charged with *N,N'*-bis(2,6-diisopropylphenyl)ethylenediimine (22.60 g, 60 mmol) and THF (300 mL). The resulting yellow solution was cooled to 0 °C in an ice/water bath before sodium borohydride (9.08 g, 240 mmol) was added in one portion. Next, concentrated hydrochloric acid (12.15 g, 10.1 mL, 120 mmol) was added dropwise in 30 min and the reaction mixture was further stirred at 0 °C for 1 h. Cold 3 M aqueous hydrochloric acid prepared by diluting 120 mL of concentrated hydrochloric acid into 360 g of ice (480 mL, 1.44 mol) was added dropwise to the resulting white suspension maintained at 0 °C in an ice/water bath. The resulting yellow suspension was warmed up slowly to room temperature and further stirred overnight. The white precipitate was filtered through a Büchner funnel and rinsed with water (3 × 100 mL). It was spread in an evaporating dish and dried until constant weight under an IR lamp to give a white powder that was pure enough for the next step (typical yield: 21.8 g, 80% yield).

Analytical data: Mp 216–218 °C (dec.). ^{1}H NMR (400 MHz, DMSO-d_6, 333 K): δ = 7.24 (m, 6 H, CH_{ar}), 4.32 (br s, NH_2^+ + H_2O), 3.45 (s, 4 H, CH_2N) 3.43 (sept, $^3J_{H,H}$ = 8.0 Hz, 4 H, C*H*(CH_3)$_2$), 1.19 (d, $^3J_{H,H}$ = 8.0 Hz, 24 H, CH(C*H*$_3$)$_2$) ppm. ^{13}C NMR (100 MHz, DMSO-d_6, 333 K): δ = 142.7 (*o*-C Dip), 134.7 (*i*-C Dip), 127.3 (*p*-CH Dip), 124.5 (*m*-CH Dip), 49.3

(CH_2N), 26.9 (CH(CH_3)$_2$), 24.2 (CH(CH$_3$)$_2$) ppm. These NMR data matched those reported in the literature.[8]

NMR spectra:

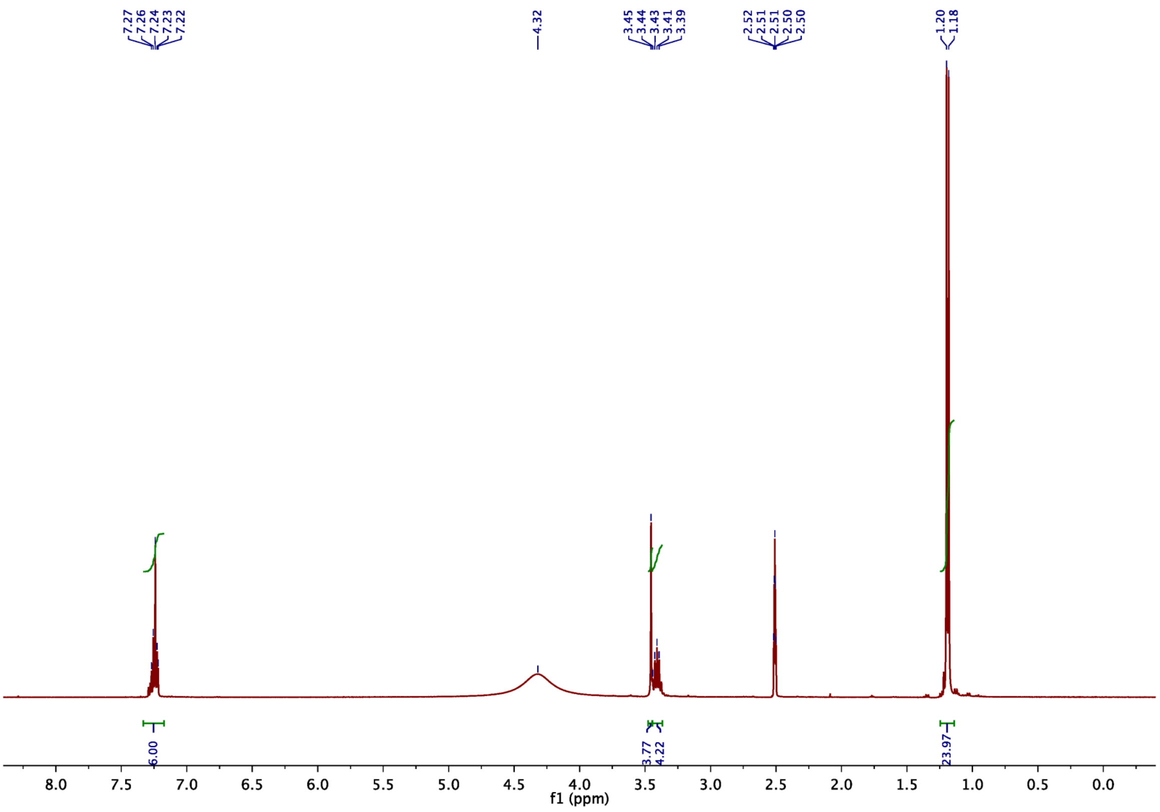

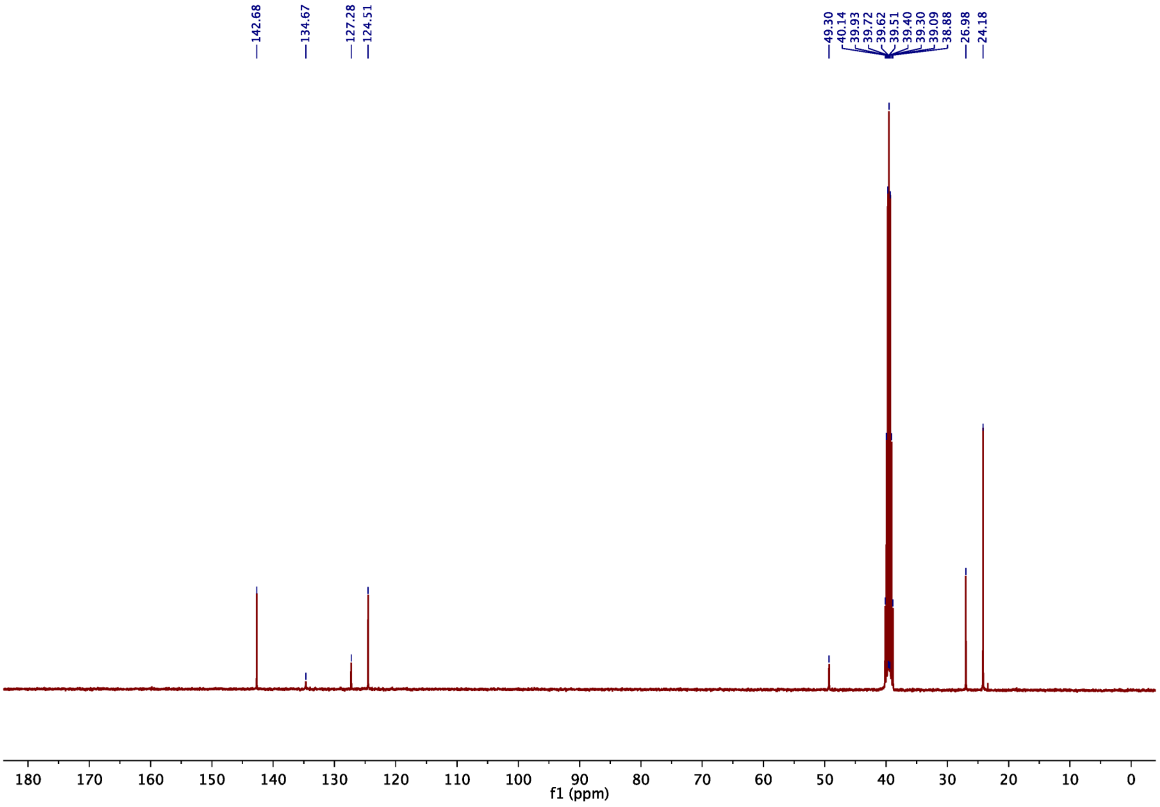

12. Synthesis of 1,3-bis(2,6-diisopropylphenyl)imidazolinium chloride (SIDip·HCl)

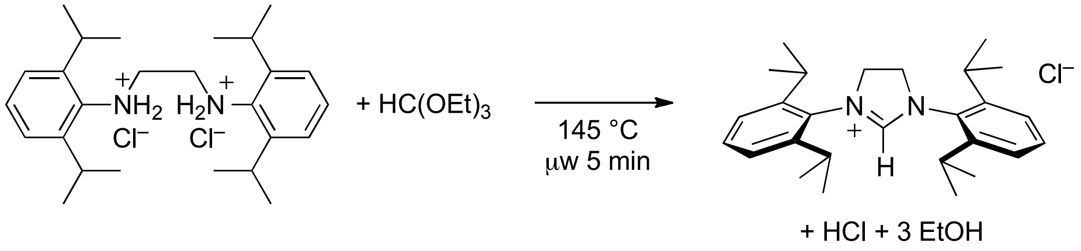

MF	$C_{26}H_{42}Cl_2N_2$	$C_7H_{16}O_3$	$C_{27}H_{39}ClN_2$
MW	453.5372	148.2016	427.0715
amount (mmol)	50	150	50
mass (g)	22.6769	22.2302	21.3536 (100% yield)

Experimental procedure: A 80 mL pressure vial equipped with a magnetic stirring bar was charged with *N,N'*-bis(2,6-diisopropylphenyl)ethylenediamine dihydrochloride (22.68 g, 50 mmol) and triethyl orthoformate (22.23 g, 25 mL, 150 mmol). The mixture was triturated with a spatula until a homogeneous thick white paste was obtained. The vial was closed with a screw cap equipped with a pressure sensor and a glass inlet tube ended by a sapphire window containing an optical fiber temperature sensor. The reaction mixture was heated for 5 min at 145 °C in a monomodal microwave reactor. The maximum microwave power was set to 150 W and the pressure limit at 250 psi. No ramp and no simultaneous cooling were applied. After rapid air cooling by the unit, the resulting beige-brown suspension was poured into a 250 mL beaker placed in an ice bath and the reaction vessel was rinsed twice with diethyl ether (2 × 50 mL). The beige suspension was filtered on a Büchner funnel and the white precipitate was rinsed with diethyl ether (2 × 50 mL). It was spread in an evaporating dish and dried until constant weight under an IR lamp to afford pure 1,3-bis(2,6-diisopropylphenyl)imidazolinium chloride as a white microcrystalline powder (typical yield: 15 g, 70% yield).

Analytical data: Mp 233–237 °C (lit. 237–240 °C).[8] ^{1}H NMR (400 MHz, DMSO-d_6): δ = 9.63 (s, 1 H, CH^2 Im), 7.56 (t, $^3J_{H,H}$ = 7.8 Hz, 2 H, *p*-CH Dip), 7.43 (d, $^3J_{H,H}$ = 8.0 Hz, 4 H, *m*-CH Dip), 4.57 (s, 4 H, $CH_2^{4,5}$ Im), 3.10 (sept, $^3J_{H,H}$ = 6.8 Hz, 4 H, C*H*(CH$_3$)$_2$), 1.36 (d, $^3J_{H,H}$ = 6.4 Hz, 12 H, CH(C*H*$_3$)$_2$), 1.20 (d, $^3J_{H,H}$ = 6.8 Hz, 12 H, CH(C*H*$_3$)$_2$) ppm. ^{13}C NMR (100

MHz, DMSO-d_6): δ = 160.1 (CH^2 Im), 146.1 (*o*-C Dip), 131.1 (*p*-CH Dip), 129.9 (*i*-C Dip), 124.8 (*m*-CH Dip), 53.7 ($CH_2^{4,5}$ Im), 28.3 ($CH(CH_3)_2$), 25.1 ($CH(CH_3)_2$), 23.3 ($CH(CH_3)_2$) ppm.

NMR spectra:

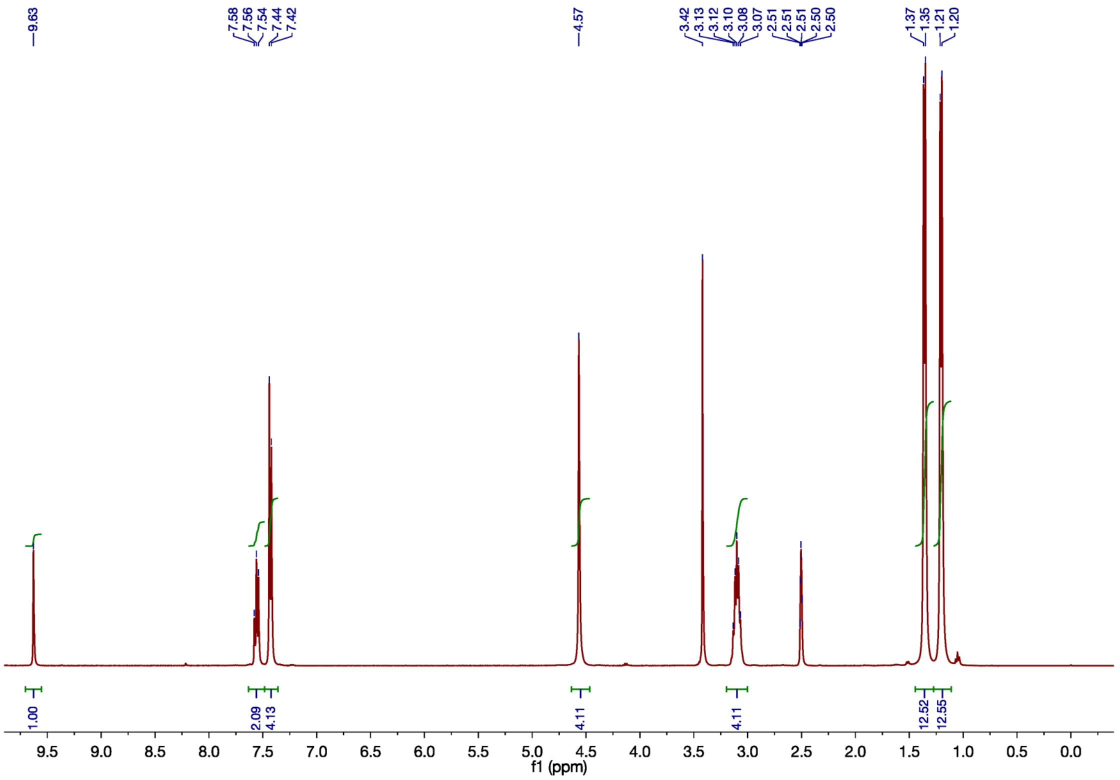

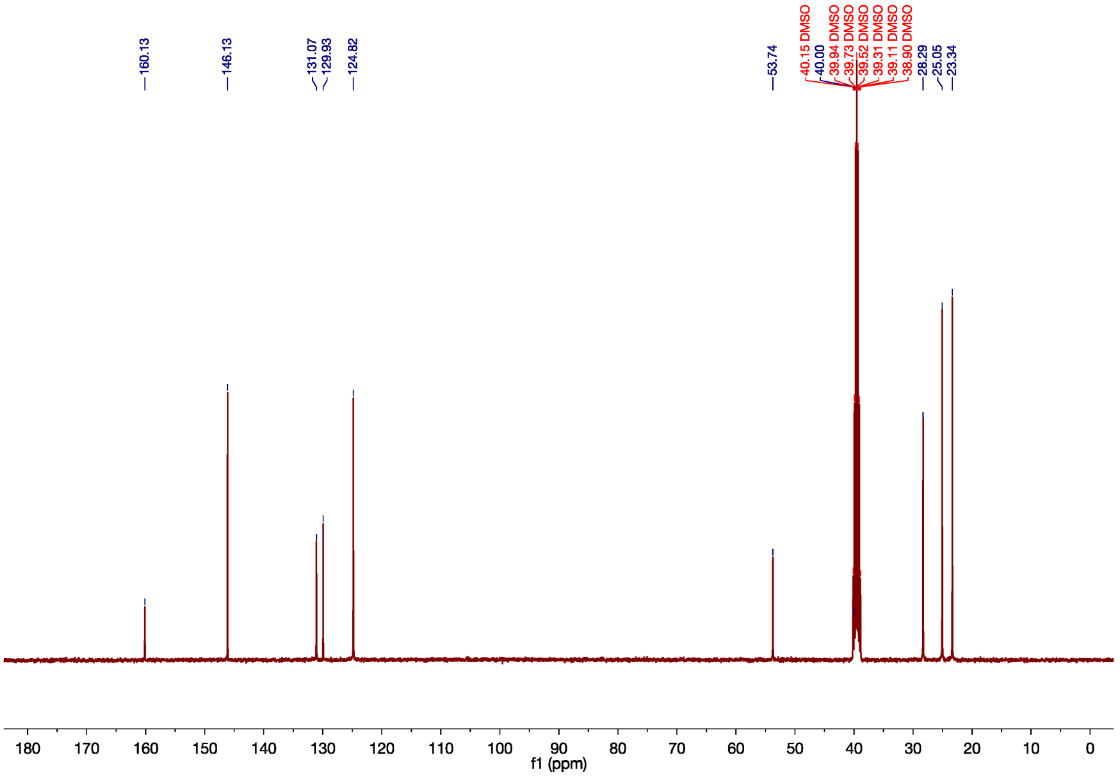

13. Synthesis of 2,6-bis(diphenylmethyl)-4-methylaniline

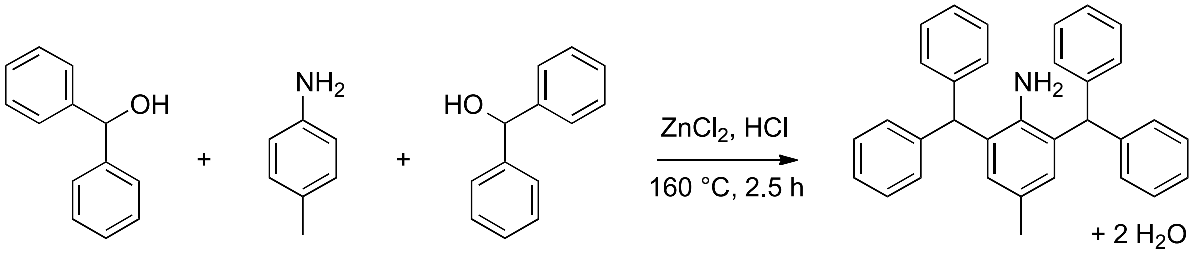

MF	$C_{13}H_{12}O$	C_7H_9N	$ZnCl_2$	HCl	$C_{33}H_{29}N$
MW	184.2372	107.1548	136.3150	36.4609	439.5988
amount (mmol)	200	100	50	100	100
mass (g)	36.8474	10.7155	6.8230	10.1280 g of 36% aq. sln	43.9599 (100% yield)

Experimental procedure: A 250 mL round-bottom flask equipped with a magnetic stirring bar but no stopper was charged with diphenylmethanol (36.85 g, 200 mol) and *p*-toluidine (10.72 g, 100 mol). The solid mixture was heated in an oil bath at 60 °C until a brown-black liquid was obtained. A colorless solution of anhydrous zinc chloride (6.81 g, 50 mol) in concentrated hydrochloric acid (36% in H_2O, 8.6 mL, 100 mol) was added dropwise to the eutectic melt. The temperature of the oil bath was then increased to 160 °C and the reaction mixture was further stirred for 1 h in the open vessel. After cooling to room temperature, the resulting ocher-brown viscous oil turned into a glassy solid that was taken up with dichloromethane (2 × 125 mL) and transferred into a 500 mL separatory funnel. The organic solution was washed with water (3 × 125 mL) and dried over anhydrous K_2CO_3. Silica gel (25 g) was added and the resulting suspension was filtered by gravity. The dark yellow filtrate was concentrated on a rotary evaporator and the remaining solid was dried until constant weight under an IR lamp to afford 2,6-bis(diphenylmethyl)-4-methylaniline as an off-white powder that was pure enough for the next step (typical yield: 37.4 g, 85% yield).

Analytical data: Mp 183–185 °C (lit. 182–183 °C).[9] ^{1}H NMR (400 MHz, $CDCl_3$): δ = 7.23–7.27 (m, 8 H, Ph), 7.17–7.21 (m, 4 H, Ph), 7.07–7.10 (m, 8 H, Ph), 6.39 (s, 2 H, *m*-CH), 5.44 (s, 2 H, C*H*Ph_2), 3.26 (s, 2 H, NH_2), 2.00 (s, 3 H, *p*-CH_3) ppm. ^{13}C NMR (100 MHz, $CDCl_3$): δ = 142.9 (*i*-C Ph), 139.8 (CNH_2), 129.7 (*m*-CH Ph), 129.3 (*C*$CHPh_2$), 129.2 (*m*-CH), 128.6 (*o*-CH Ph), 126.74 (*C*CH_3), 126.69 (*p*-CH Ph), 52.5 ($CHPh_2$), 21.1 (*p*-CH_3) ppm.

NMR spectra:

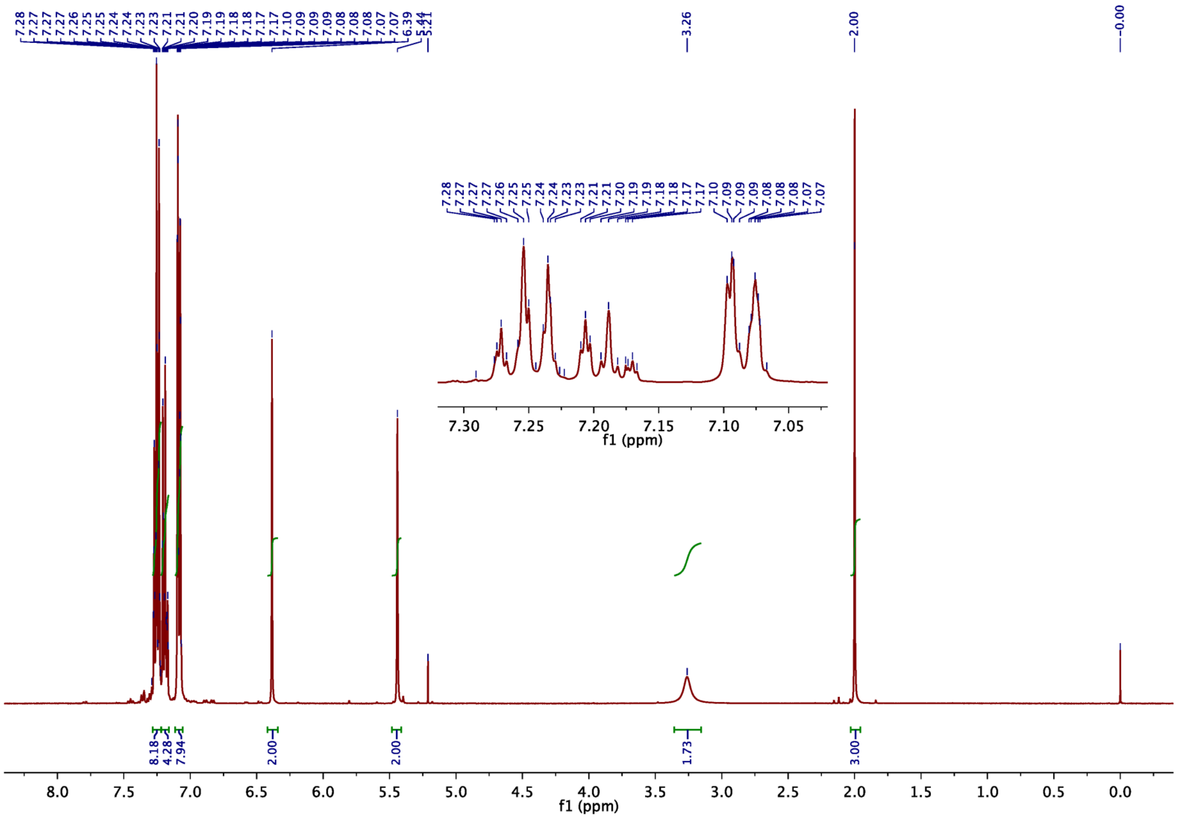
7.28
7.27
7.27
7.27
7.26
7.25
7.25
7.24
7.24
7.23
7.23
7.21
7.21
7.20
7.19
7.19
7.18
7.18
7.17
7.17
7.10
7.09
7.09
7.09
7.09
7.08
7.08
7.08
7.07
7.07
6.39
5.44
5.21
3.26
2.00
-0.00
8.18
4.28
7.94
2.00
2.00
1.73
3.00
f1 (ppm)

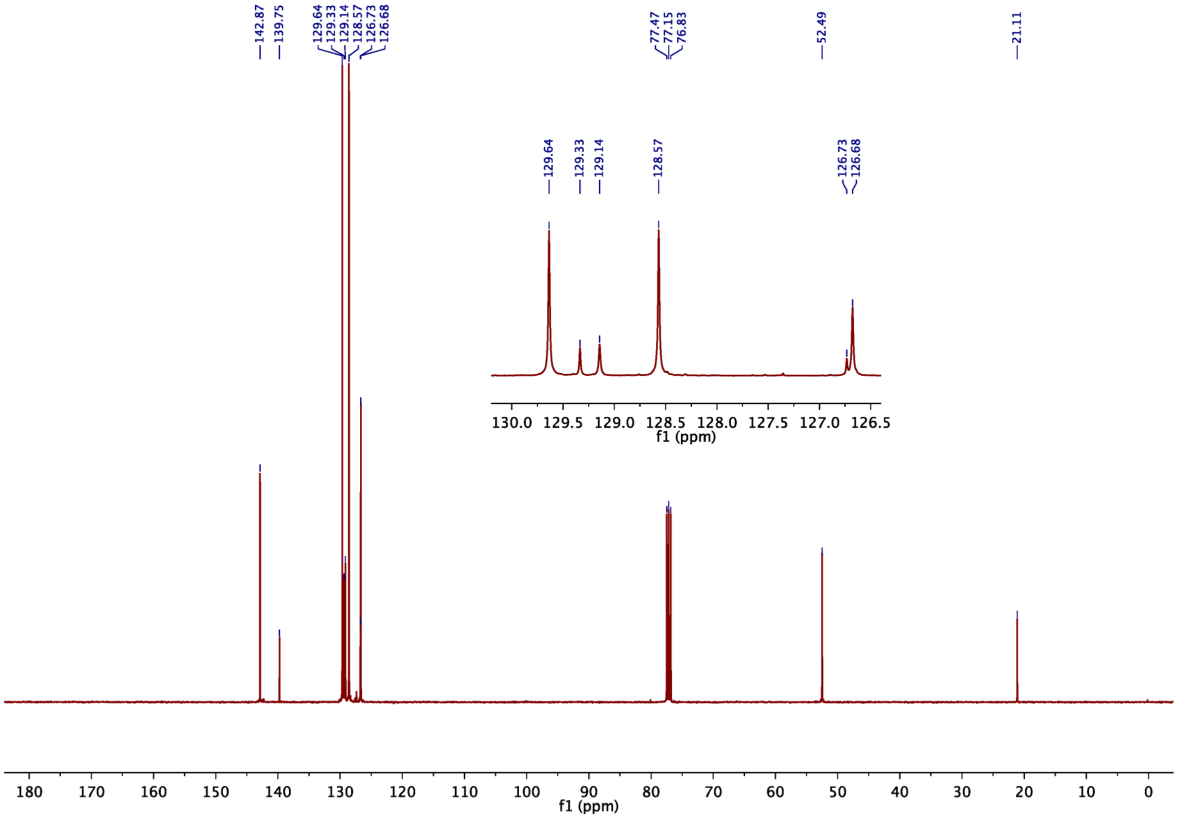
142.87
139.75
129.64
129.33
129.14
128.57
126.73
126.68
77.47
77.15
76.83
52.49
21.11
f1 (ppm)

14. Synthesis of *N,N'*-bis(2,6-bis(diphenylmethyl)-4-methylphenyl)ethylene-diimine

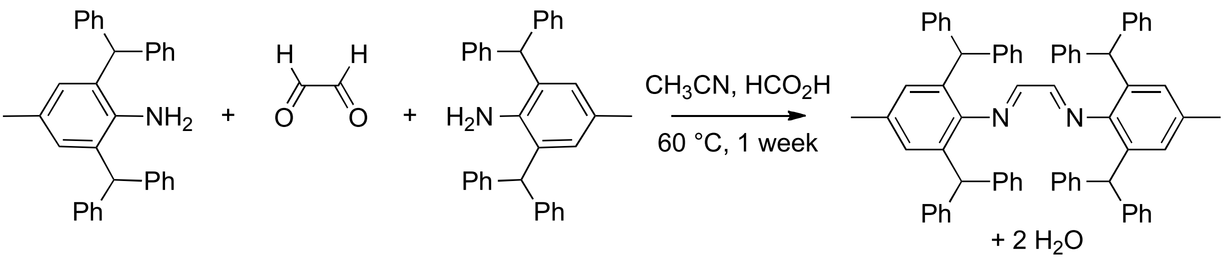

MF	$C_{33}H_{29}N$	$C_2H_2O_2$	$C_{68}H_{56}N_2$
MW	439.5988	58.0366	901.2038
amount (mmol)	40	20	20
mass (g)	17.5840	2.9018 g of 40% aqueous solution	18.0241 (100% yield)

Experimental procedure: A 500 mL round-bottom flask equipped with a magnetic stirring bar was charged with 2,6-bis(diphenylmethyl)-4-methylaniline (17.58 g, 40 mmol) and acetonitrile (400 mL). Glyoxal (40 % aqueous solution, 2.90 g, 2.3 mL, 20 mmol) and formic acid (6 drops) were added and the white suspension was stirred for 7 days in an oil bath at 60 °C. The resulting yellow suspension was cooled to room temperature and filtered on a Büchner funnel. The yellow precipitate was rinsed with a small portion of acetonitrile. It was spread in an evaporating dish and dried until constant weight under an IR lamp to afford *N,N'*-bis(2,6-bis(diphenylmethyl)-4-methylphenyl)ethylenediimine as a yellow powder that was pure enough for the next step (typical yield: >13.5 g, >75% yield).[3]

Analytical data: Mp 237–238 °C. ^{1}H NMR (400 MHz, $CDCl_3$): δ = 7.13–7.22 (m, 26 H, Ph + CH=N), 6.97 (d, $^{3}J_{H,H}$ = 7.2 Hz, 16 H, Ph), 6.65 (s, 4 H, *m*-CH), 5.23 (s, 4 H, C*H*Ph_2), 2.10 (s, 6 H, *p*-CH_3) ppm. ^{13}C NMR (100 MHz, $CDCl_3$): δ = 164.0 (CH=N), 147.1 (*i*-C), 144.1 (*i*-C Ph), 133.7 (*C*CH_3), 132.2 (*C*$CHPh_2$), 129.7 (*m*-CH Ph), 129.3 (*m*-CH), 128.4 (*o*-CH Ph), 126.4 (*p*-CH Ph), 51.3 ($CHPh_2$), 21.4 (*p*-CH_3) ppm. These NMR data matched those reported in the literature.[9]

NMR spectra:

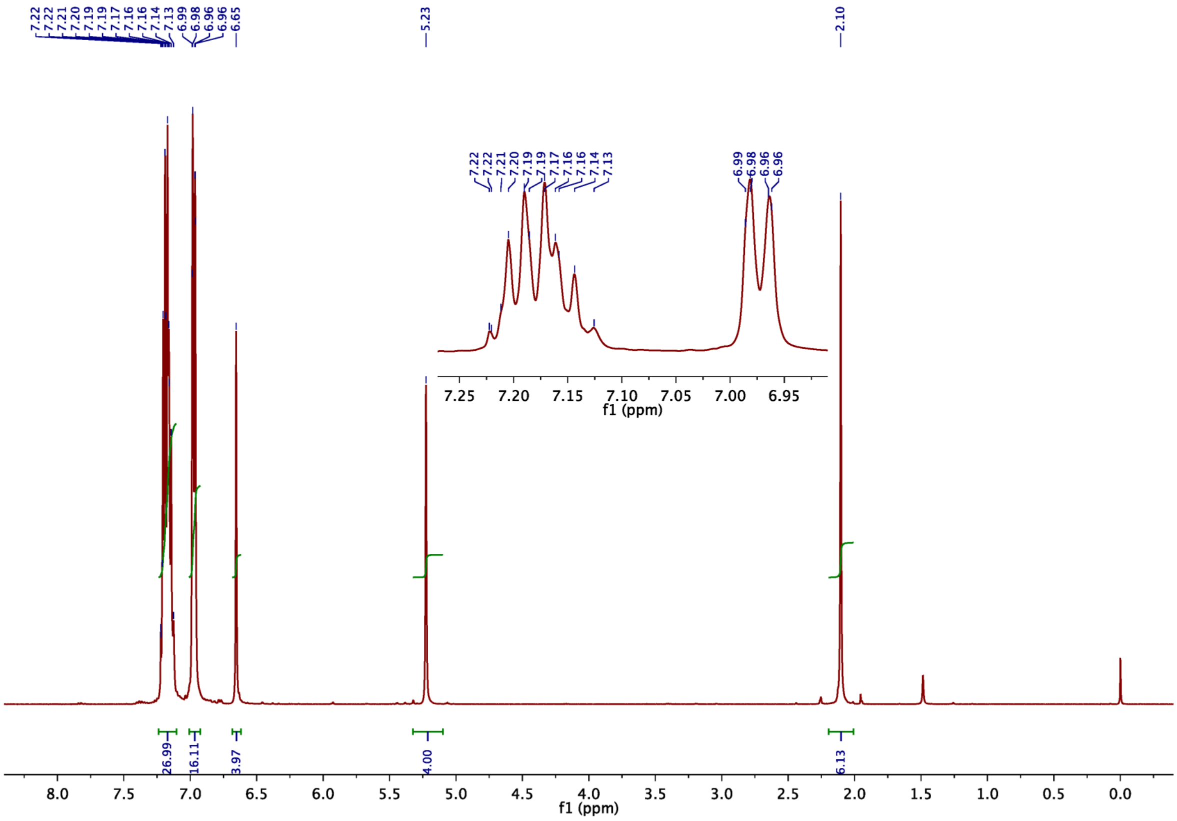
7.22 7.22 7.21 7.20 7.19 7.19 7.17 7.16 7.16 7.14 7.13 6.99 6.98 6.96 6.96 6.65
5.23
2.10
26.99 16.11 3.97 4.00 6.13
f1 (ppm)

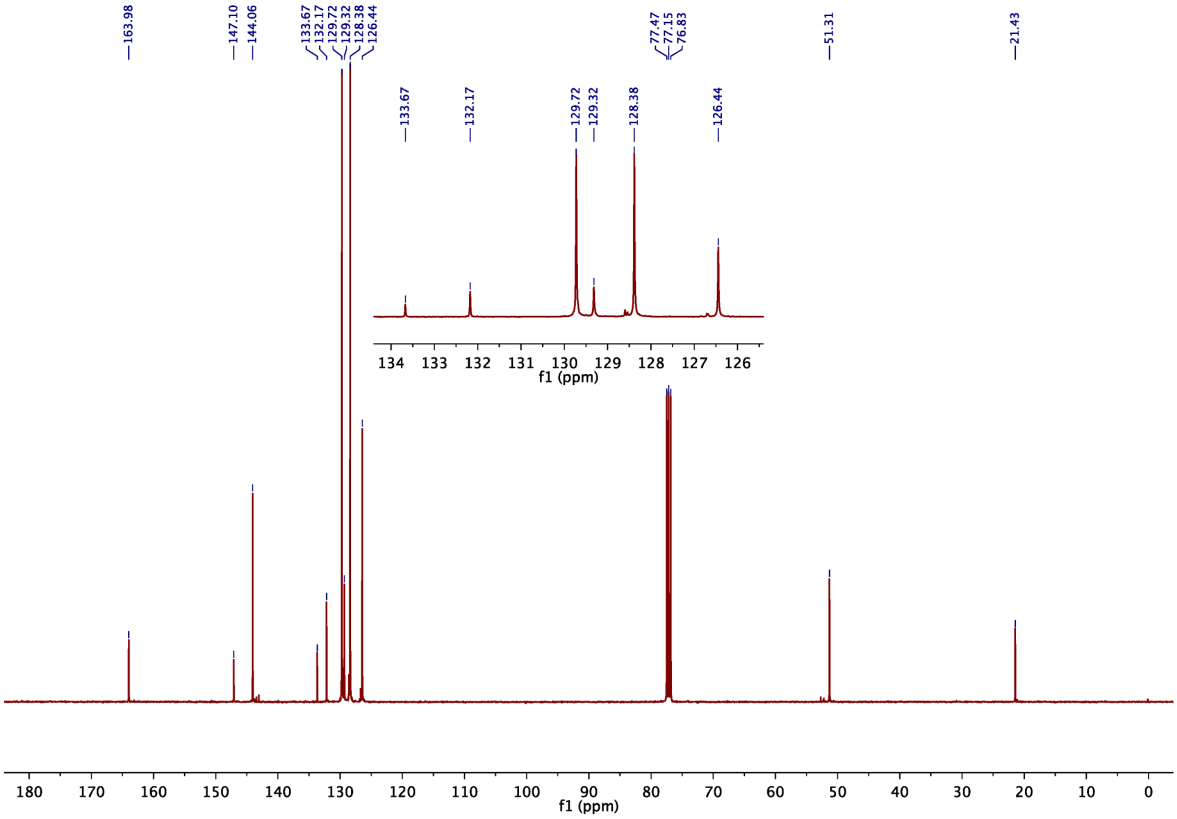
163.98
147.10 144.06
133.67 132.17 129.72 129.32 128.38 126.44
77.47 77.15 76.83
51.31
21.43
f1 (ppm)

15. Synthesis of 1,3-bis(2,6-bis(diphenylmethyl)-4-methylphenyl)-imidazolium chloride (IDip*·HCl)

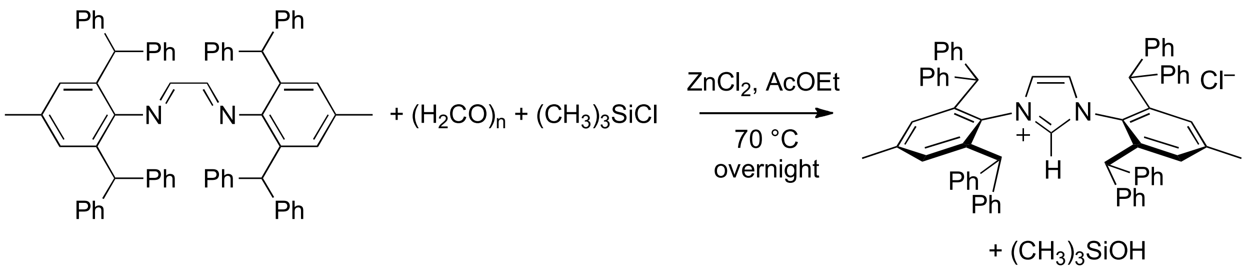

MF	$C_{68}H_{56}N_2$	$ZnCl_2$	$(CH_2O)_n$	C_3H_9SiCl	$C_{69}H_{57}ClN_2$
MW	901.2038	136.3150	30.0262	108.6426	949.6757
amount (mmol)	10	10	10	10	10
mass (g)	9.0120	1.3632	0.3003	1.0864	9.4968 (100% yield)

Experimental procedure: A 100 mL round-bottom flask equipped with a magnetic stirring bar was charged with *N,N'*-bis(2,6-bis(diphenylmethyl)-4-methylphenyl)ethylenediimine (10 mmol), anhydrous zinc chloride (1.36 g, 10 mmol), paraformaldehyde (0.30 g, 10 mmol), and chloroform (60 mL). The suspension was heated in an oil bath at 60 °C until a clear dark red solution was obtained. The solvent was removed on a rotary evaporator and the residue was taken up with ethyl acetate (50 mL). Next, a solution of chlorotrimethylsilane (1.09 g, 1.3 mL, 10 mmol) in ethyl acetate (10 mL) was added dropwise at room temperature. The resulting yellow-orange solution was further stirred overnight in an oil bath at 70 °C. The color changed to black-brown and the mixture was cooled to room temperature. It was transferred into a 250 mL separatory funnel and washed twice with water (2 × 50 mL) and once with brine (50 mL). The organic phase was dried over $MgSO_4$ and the solvent was removed on a rotary evaporator. The resulting brown solid was recrystallized from dichloromethane/ diethylether to afford pure 1,3-bis(2,6-bis(diphenylmethyl)-4-methylphenyl)imidazolium chloride as an off-white microcrystalline powder (typical yield: 6.2 g, 65% yield).

Analytical data: Mp 316–317 °C (dec.). ^{1}H NMR (400 MHz, $CDCl_3$): $\delta = 12.96$ (s, 1 H, CH^{2} Im), 7.10–7.28 (m, 32 H, Ph), 6.77–6.80 (m, 12 H, *m*-CH + Ph), 5.48 (s, 2 H, $CH^{4,5}$ Im), 5.28 (s, 4 H, C*H*Ph_2), 2.19 (s, 6 H, *p*-CH_3) ppm. ^{13}C NMR (100 MHz, $CDCl_3$): $\delta = 142.5$ (C_{ar}),

141.9 (C_{ar}), 141.5 (C_{ar}), 140.6 (C_{ar}), 130.8 (*m*-CH), 130.1 (CH_{ar}), 130.0 (CH^2 Im), 129.2 (CH_{ar}), 128.9 (CH_{ar}), 128.7 (CH_{ar}), 127.1 (CH_{ar}), 126.9 (CH_{ar}), 123.5 ($CH^{4,5}$ Im), 51.4 ($CHPh_2$), 21.9 (*p*-CH_3) ppm. These NMR data matched those reported in the literature.[9]

NMR spectra:

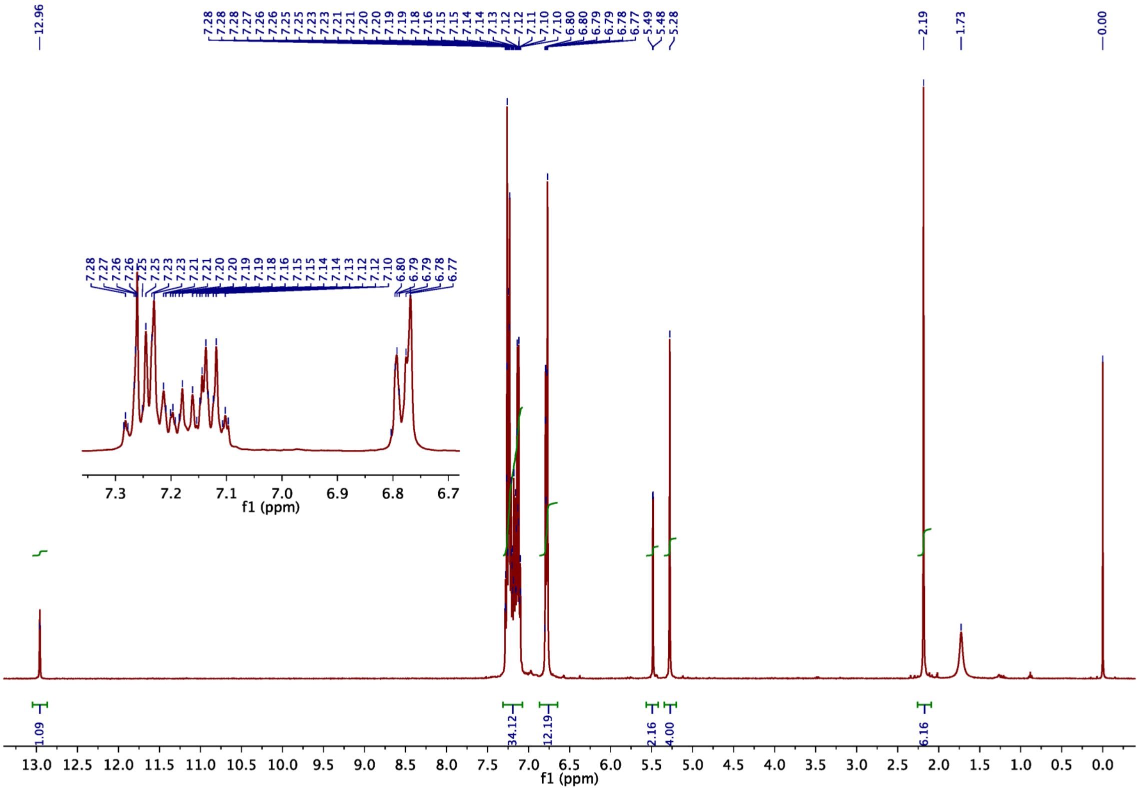

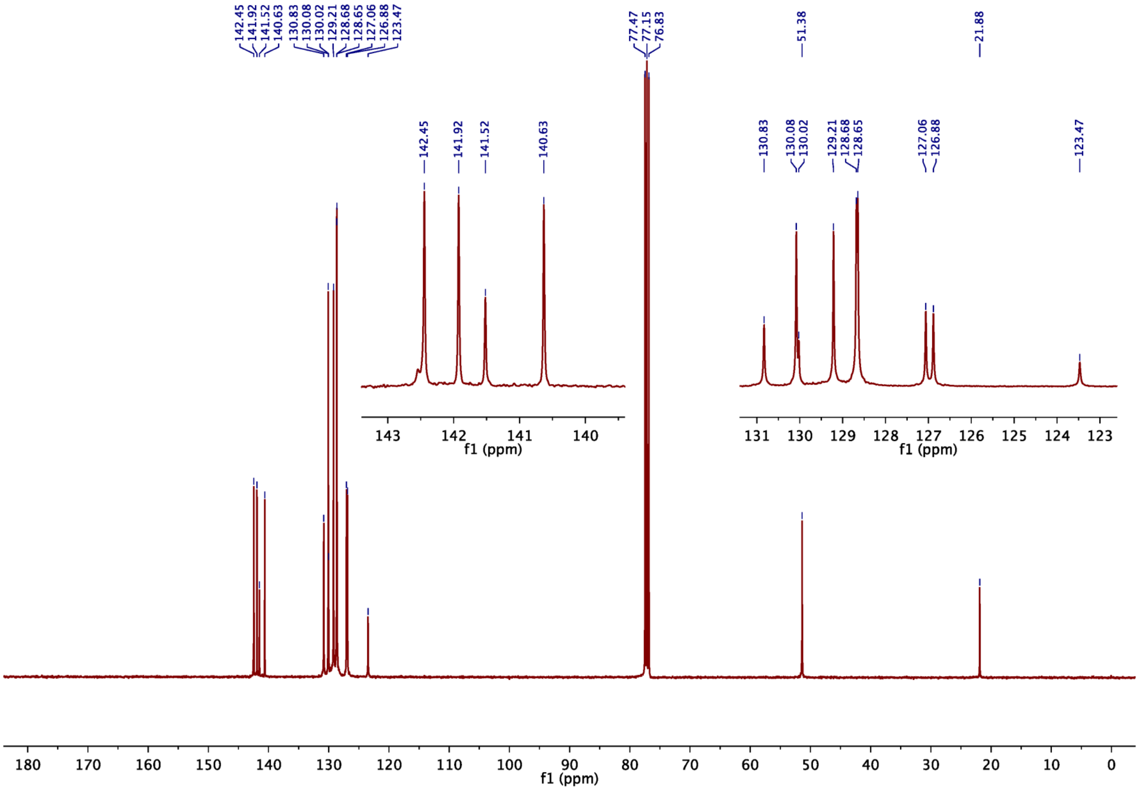

16. References and notes

1. Hans, M.; Wouters, J.; Demonceau, A.; Delaude, L. *Chem. Eur. J.* **2015**, *21*, 10870–10877.

2. Chan, K.-T.; Tsai, Y.-H.; Lin, W.-S.; Wu, J.-R.; Chen, S.-J.; Liao, F.-X.; Hu, C.-H.; Lee, H. M. *Organometallics* **2010,** *29*, 463–472.

3. Typical yield for the first crop of diimine. Second and even third crops of product usually precipitated from the mother liquor. They may be added to the first crop in order to further increase the yield, but their purity needs to be checked beforehand.

4. Hans, M.; Delaude, L. *Org. Synth.* **2010,** *87*, 77–87.

5. Hintermann, L. *Beilstein J. Org. Chem.* **2007,** *3*, No. 22.

6. Hans, M.; Wouters, J.; Demonceau, A.; Delaude, L. *Chem. Eur. J.* **2013,** *19*, 9668–9676.

7. Nolte, C.; Mayer, P.; Straub, B. F. *Angew. Chem. Int. Ed.* **2007**, *46*, 2101–2103.

8. Arduengo, A. J., III; Krafczyk, R.; Schmutzler, R.; Craig, H. A.; Goerlich, J. R.; Marshall, W. J.; Unverzagt, M. *Tetrahedron* **1999,** *55*, 14523–14534.

9. Berthon-Gelloz, G.; Siegler, M. A.; Spek, A. L.; Tinant, B.; Reek, J. N. H.; Markó, I. E. *Dalton Trans.* **2010,** *39*, 1444–1446.
